# Comprehensive bioinformatics and machine learning analyses for breast cancer staging using TCGA dataset

**DOI:** 10.1093/bib/bbae628

**Published:** 2024-12-04

**Authors:** Saurav Chandra Das, Wahia Tasnim, Humayan Kabir Rana, Uzzal Kumar Acharjee, Md Manowarul Islam, Rabea Khatun

**Affiliations:** Department of Computer Science and Engineering, Jagannath University, Dhaka-1100, Bangladesh; Department of Internet of Things and Robotics Engineering, Bangabandhu Sheikh Mujibur Rahman Digital University, Bangladesh, Kaliakair, Gazipur-1750, Bangladesh; Department of Computer Science and Engineering, Green University of Bangladesh, Narayanganj-1461, Dhaka, Bangladesh; Department of Computer Science and Engineering, Green University of Bangladesh, Narayanganj-1461, Dhaka, Bangladesh; Department of Computer Science and Engineering, Jagannath University, Dhaka-1100, Bangladesh; Department of Computer Science and Engineering, Jagannath University, Dhaka-1100, Bangladesh; Department of Computer Science and Engineering, Green University of Bangladesh, Narayanganj-1461, Dhaka, Bangladesh

**Keywords:** breast cancer, TCGA, cancer staging, ontology, machine learning, transcription factors

## Abstract

Breast cancer is an alarming global health concern, including a vast and varied set of illnesses with different molecular characteristics. The fusion of sophisticated computational methodologies with extensive biological datasets has emerged as an effective strategy for unravelling complex patterns in cancer oncology. This research delves into breast cancer staging, classification, and diagnosis by leveraging the comprehensive dataset provided by the The Cancer Genome Atlas (TCGA). By integrating advanced machine learning algorithms with bioinformatics analysis, it introduces a cutting-edge methodology for identifying complex molecular signatures associated with different subtypes and stages of breast cancer. This study utilizes TCGA gene expression data to detect and categorize breast cancer through the application of machine learning and systems biology techniques. Researchers identified differentially expressed genes in breast cancer and analyzed them using signaling pathways, protein–protein interactions, and regulatory networks to uncover potential therapeutic targets. The study also highlights the roles of specific proteins (MYH2, MYL1, MYL2, MYH7) and microRNAs (such as hsa-let-7d-5p) that are the potential biomarkers in cancer progression founded on several analyses. In terms of diagnostic accuracy for cancer staging, the random forest method achieved 97.19%, while the XGBoost algorithm attained 95.23%. Bioinformatics and machine learning meet in this study to find potential biomarkers that influence the progression of breast cancer. The combination of sophisticated analytical methods and extensive genomic datasets presents a promising path for expanding our understanding and enhancing clinical outcomes in identifying and categorizing this intricate illness.

## Introduction

Breast cancer is the second most common cancer among women, following skin cancer, and is the second leading cause of cancer-related mortality, after lung cancer [1]. Globally, breast cancer has surpassed lung cancer as the most frequently diagnosed cancer in women. In 2020, an estimated 2 261 419 women worldwide were diagnosed with breast cancer [2]. According to the American Society for Clinical Oncology, it is projected that in 2023, 297 790 women in the USA will be diagnosed with invasive breast cancer, while 55 720 will be diagnosed with noninvasive (*in situ*) breast cancer. Since the mid-2000s, the incidence of invasive breast cancer in women has increased by ~0.5% annually, likely driven by factors such as declining fertility rates, delayed age of first childbirth, and rising obesity rates. Additionally, invasive breast cancer is expected to affect ~2800 men in the USA in 2023 [3]. Early detection of breast cancer is critical for selecting appropriate treatments and reducing the risk of metastasis [4]. Breast cancer is a heterogeneous and evolving disease, marked by various somatic mutations and changes in gene and protein expression. It is classified into several subtypes based on the expression of the progesterone receptor (PR), estrogen receptor (ER), and human epidermal growth factor receptor 2 (HER2). Each subtype requires specific treatment approaches, which can affect drug resistance, cancer recurrence, and mortality rates [5, 6]. Identifying novel clinical biomarkers is essential for better patient stratification; enhancing the accuracy of initial diagnoses; and monitoring the progression, metastasis, and recurrence of breast cancer [7].

In present days, tumor markers have become increasingly prevalent in areas of cancer detection and therapy. For tumor screening, diagnosis, efficacy and prognosis evaluation, recurrence detection, and so forth, the optimal tumor marker should possess high specificity and the ability to recognize tiny lesions and quantify the tumor burden [8]. Staging a cancer is the process of quantifying the extent of the cancer’s metastasis throughout the body. The process of measuring and evaluating the extent to which cancer has progressed to various sections of the body is referred to as cancer staging. It is helpful in choosing the most efficient kind of therapy as well as detecting the degree to which the cancer has spread. Additionally, it is used by physicians in the process of calculating survival rates. The Joint Working Committee for Cancer Tumor-Lymph Node-Metastasis states that there are typically five different stages of cancer: Stages 0, I, II, III, and IV [9].In addition to determining the cancer’s size and location, the stage of the disease will also impact the existence of indicators of cancer spread and how much cancer has progressed to neighboring tissues, lymph nodes, and other body regions [10]. For individuals between the ages of 18 and 55 who suffer from breast cancer, with the greatest grade detected at stage I, the 5-year survival rate is 97% and can be cured with the right care, whereas the projected 5-year survival rates of stages II, III, and IV are 92%, 77%, and 28%, respectively [11]. Among all cancers, breast cancer mutations are the most prevalent and lethal. A patient’s chances of survival are significantly increased when the illness is identified in its early stages [12].

Machine learning could uncover correlations that are difficult to recognize in vast, noisy, or complicated datasets. This skill is specifically appropriate for data analysis applications in the healthcare sector, particularly those requiring intricate proteomics and genomic expression–based applications, which have been employed commonly in recent years for the identification and treatment of cancer [13]. In the medical area, machine learning techniques are commonly employed including random forests (RFs) [14], support vector machine (SVM) [15–17], and decision trees (DTs) [18, 19]. Applications like Xie *et al*.’s [20] employed spectral data to create SVM models with an average accuracy of 100% for quick and noninvasive keratitis detection, and SVM and DT models were utilized by Chen *et al*. [21] to quickly detect gliomas with a predictive accuracy of ~90%. It demonstrates even more that machine learning is more applicable to the diagnosis of diseases. A supervised learning model called SVM is capable of handling both linear and nonlinear problems. It works to address issues with classification and regression. The ultimate class of a test object is determined by combining a collection of DTs that are randomly chosen from the training set. RFs are a powerful force in machine learning because DTs, which are renowned for their adaptability, excel in classification and regression tasks.

In the field of biomedicine, there have been some encouraging results using gene network–based cancer prediction and biomarker screening. Jubair *et al*.’s [22] subtype-specific network biomarker approach, for example, has demonstrated high predictive effectiveness for identifying the survivorship of breast cancer patients with it. Li *et al*. [23] constructed a model to predict the prognosis of cervical cancer patients using the weighted gene coexpression network paired alongside the Least Absolute Shrinkage and Selection Operator (LASSO) technique and showed that the approach is legitimate and reliable. In this work, we first performed a differential expression analysis between breast cancer and healthy controls. The LASSO feature selection approach has been employed in a variety of biological applications. Lasso is a well-known feature selection approach that takes into account an L1 type penalty, which places a restriction on the combined value of all absolute values for the feature parameters to ensure global optimal performance as well as computing efficiency [24]. The 2021 IEEE International Conference on Bioinformatics and Biomedicine found that LASSO consistently beats other methods in several key classification parameters, especially the area under the curve (AUC), and that the LASSO framework can generate more meaningful feature selection algorithms relative to similar feature selection methods for features [25]. Furthermore, Maurya *et al*. [26] effectively utilized the Lasso algorithm in the field of cancer by extracting signature genes by LASSO and other techniques, leading to the discovery of TMEM236—a new biomarker for the detection of colorectal cancer. Additionally, two separate groups of the first three stages of breast cancer were identified to carry out the differential expression analysis: the first stage versus the subsequent three phases and early-stage disease versus advanced or metastatic cancer, respectively [27]. Depending on whether the breast cancer had spread to nearby lymph nodes or somewhere else, it was split into two groups. For the staging groups, differential expression analysis was performed. Following PPI analysis, the final feature genes used for classification were searched for prognostic genes. Breast cancer and breast cancer staging were finally classified using machine learning algorithms such as the SVM, RF, and DTs. In this case, the results of the model constructed using the features we extracted produced better results for the early and late diagnosis of breast cancer, and the prognostic genes that were examined provided further recommendations for the treatment of breast cancer.

This work presents a new strategy in breast cancer research by combining systems biology methods and machine learning algorithms—an unusual combination. In addition to providing a greater understanding of the molecular pathways underlying the disease, this dual approach improves the accuracy of cancer diagnosis and staging. Furthermore, the discovery of certain proteins (MYH2, MYL1, MYL2, MYH7) and microRNAs (hsa-let-7d-5p) linked to the advancement of breast cancer offers new, prospective biomarkers for diagnosis and therapy, opening the door for innovative therapeutic approaches. The work increases our understanding of breast cancer by applying sophisticated computational algorithms to uncover stage-specific genetic markers using the extensive TCGA dataset, one of the biggest cancer datasets available. Some of the significant contributions of our work are outlined as follows:

i) Diagnosis of breast cancer using gene expression profiling data from the TCGA dataset.

ii) Establishment of protein–protein interaction (PPI) network of the differentially expressed genes (DEGs).

iii) Classification of different stages of breast cancer using the TCGA dataset with machine learning analysis.

## Materials and methods

### Workflow of the analytical approach

**Figure 1:**
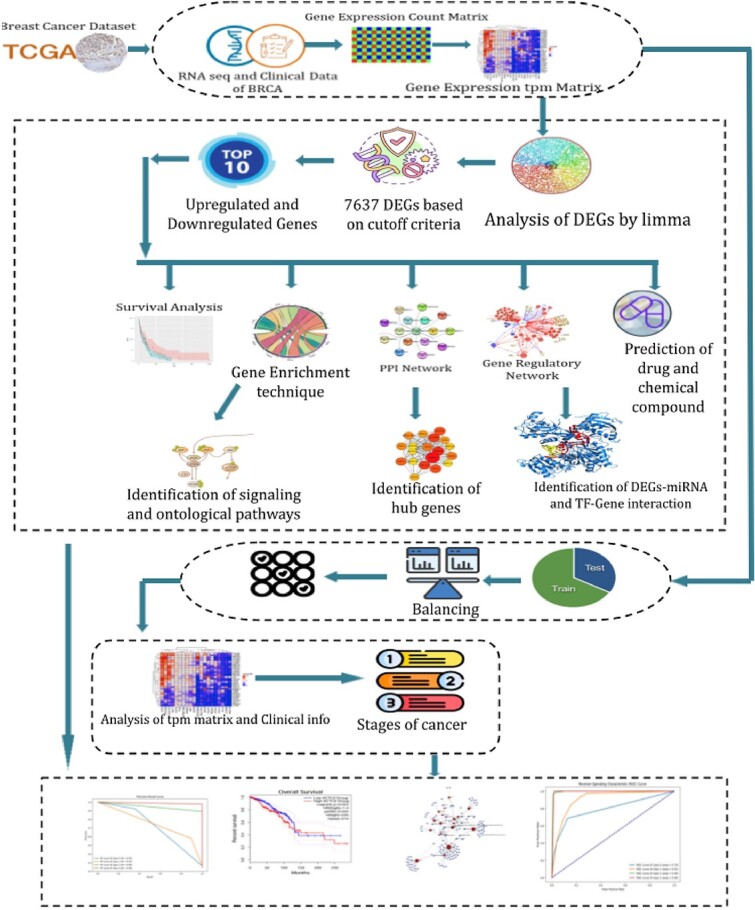
Working flowchart of the analytical study performed in this research.

To progress the work, we start by acquiring the TCGA-BRCA dataset from the Genomic Data Commons (GDC) portal; RNAseq data and clinical data from TCGA-BRCA are collected and downloaded for further progress. To find the marker genes, we performed an analytical approach presented in Fig. 1. First, we identified the DEGs in the BRCA dataset using the gene expression counts matrix. After this, some DEGs were amended based on potential DEG selection criteria, which is |logFC| > 1.0 & adj.P < 0.05. From the selected DEGs, top 10 unregulated and top 10 downregulated DEGs were used for further examination of PPIs, ontological and enrichment analysis, regulatory analysis, and prediction of drug and chemical compounds. Later on, from survival analysis, the survival curves of the most influential genes from the top 10 up and downregulated genes were found. The identification of signaling and ontological terms was fourth from gene enrichment analysis. Potential hub proteins were identified from the PPI network analysis. TFs (transcription factors) and miRNAs were identified from the gene regulatory network analysis. Furthermore, samples from the TCGA-BRCA project were employed in the experiment for the diagnosis of breast cancer and general health. Following feature extraction, important genes have been selected as diagnostic classification features. SVMs, RF, DTs, XGBoost, and AdaBoost are the classifiers used in this experiment by the researchers.

### Dataset description

We collected the TCGA-BRCA gene expression data from the GDC portal (https://portal.gdc.cancer.gov/repository). The transcriptome gene expression data from the TCGA-BRCA projects were chosen for this study, and, from these, 1224 samples of 1111 tumor tissue samples and 113 normal tissue samples were obtained for further analysis.

### Analysis of differential expression gene

Examining transcriptomic data using differential expression (DE) analysis allows for analyzing variations in gene expression across the entire genome linked to significant biological conditions [28]. DEGs play a vital role in order to gain additional biological insights, such as identifying enriched functional pathways, gene ontologies, and PPI analysis. In this study, the R package limma [29] with |logF C| > 1.0 and adj.P.Val < 0.05 was used to perform the differential expression analysis. Breast cancer tissues were compared to normal tissues to identify genes that were differentially expressed. Later on, the top 10 upregulated and downregulated genes were selected based on the logFC values from the DEGs that were used for analyzing further processes.

### Analysis of the enrichment of gene set

A computational and statistical approach known as “gene set enrichment analysis” is typically used to determine if a collection of determined genes exhibits statistical significance under various biological circumstances [30]. The structural and computational data pertaining to gene product–based functions can be found in the Gene Ontology (GO) resources [31]. Molecular function, biological process, and cellular component are the three subcategories of GO that can be used to annotate gene products [32]. In this research, the online web tool “Enrichr” was utilized for performing gene enrichment and ontology analysis. Enrichr is a user-friendly, web-based enrichment analysis application that offers a variety of visualization summaries of the combined activities of gene lists [33]. We assessed the biological relevance of the top 10 upregulated and top 10 downregulated DEGs of BRCA using signaling and gene ontology terms. In gene enrichment analysis, we selected pathways based on adjusted *P*-value <0.05. The top signaling pathways were found using three databases: Kyoto Encyclopedia of Genes and Genomes (KEGG), BioPlanet, and BioCarta. Top gene ontology terms of molecular function, biological activity, and cellular components were also identified using EnrichR.

### Identification hub-bottleneck proteins from protein–protein interaction network

Analysis of PPIs yields prominent insights into the functions of proteins and is considered the first step in systems biology and drug discovery [34]. We utilized the top 10 upregulated and downregulated DEGs of BRCA to design a PPI interaction network using the NetworkAnalyst tool. NetworkAnalyst is a web-based application that allows bench researchers to conduct both simple and complex meta-analyses of gene expression data [35]. We obtained the hub bottleneck genes from PPI analysis by using Cytohubba in Cytoscape software. Cytoscape is an open-source software framework that can integrate complicated network visualizations with any kind of attribute data [36]. Cytohubba is a Cytoscape plugin that can predict and investigate key nodes and subnetworks inside a given network [37]. In a complex PPI network, hub nodes are often identified by their extensive connectivity [38]. Hub nodes play a crucial role in both regulating several biological processes and maintaining the structural integrity of PPI networks.

### Analysis of gene regulatory networks

To understand the functions of TFs and microRNAs (miRNAs), which play a significant role in modifying the expression of DEGs linked to breast cancers, the gene regulatory network analysis was performed. Comprehensive studies are completed with the help of the online tool NetworkAnalyst [35], which utilizes databases like the TarBase [39] and miRTarBase [40] for DEG–miRNA interactions and the JASPAR database [41] for TF-DEGs interactions. The target of these experiments is to gain a clear concept of the complex transcriptional and post-transcriptional regulatory mechanisms affecting gene expression in breast cancer. Understanding the molecular mechanisms underlying the pathogenesis of breast cancer is improved by defining these regulatory relationships.

### Prediction of drugs and chemical compounds

Using the top 10 upregulated and downregulated genes of BRCA, we were able to create networks of interactions between proteins and chemicals and drugs in this analysis. The combined protein–drug and protein–chemical interactions are obtained using the NetworkAnalyst web tool. Analyzing protein–drug interactions is crucial to comprehending the structural features required for receptor sensitivity [42]. Protein–chemical interaction analysis is essential for advancing our understanding of biology, accelerating drug discovery efforts, and improving diagnostics and treatments for various diseases.

### Survival analysis

One widely used characteristic in research to predict and identify gene signatures in cancer is patient survival analysis, which combines both gene expression and clinical data [43]. The top 10 upregulated and downregulated genes from DEG analysis were subjected to survival analysis to find genes affecting breast cancer survival. Survival analysis of the top 10 upregulated and top 10 downregulated genes was performed using GEPIA2 (http://gepia2.cancer-pku.cn/#index). GEPIA2 is an upgraded web server designed for interactive assessment and large-scale gene analysis. GEPIA2 facilitates the investigation of a particular cancer subtype and subgroup comparison, extending gene expression measurement from the genetic level to the transcripts level [44].

### Building the model

In this study, 1224 samples from the TCGA-BRCA research were utilized for the diagnosis and classification of breast cancer stages. These samples included 1111 tumor tissue samples and 113 corresponding control tissue samples. The primary objective was to identify DEGs to serve as classification features, which were then used to diagnose early and late stages of breast cancer. The dataset comprised 918 samples in the early stage and 306 samples in the late stage. Early-stage breast cancer generally refers to Stages I and II and late-stage breast cancer usually encompasses Stages III and IV. Differential analysis was conducted on these samples to identify DEGs, highlighting the genes that are significantly upregulated or downregulated in breast cancer tissues compared to control tissues, providing vital information for accurate diagnosis and staging of the disease.

The study employed multiple classifiers to ensure robust and accurate predictions. These classifiers include Gaussian Naive Bayes (GNB), RF, DT, *K*-Nearest Neighbors (KNNs), XGBoost, and SVM with RBF Kernel Function. To guarantee that the features were on a similar scale, each classifier started by normalizing the data. This is an important step for enhancing performance, particularly for algorithms that are sensitive to data scale. The dataset was split at random into a test set (30%) and a training set (70%) to give enough information for learning while keeping a sizeable amount for objective assessment. The Synthetic Minority Over-sampling Technique (SMOTE) is used to solve the problem of sample imbalance, particularly between the early and late stages of breast cancer. To balance the class distribution and improve the model’s capacity to generalize and perform well in minority classes, SMOTE creates synthetic examples for the training set. The test set data were used to assess the trained models, and the performance metric was the AUC of the receiver operating characteristic (ROC) curve. A higher AUC denotes greater performance. The AUC gives an indication of how well the model can discriminate across classes. The outcomes were averaged across 10 runs using cross-validation, a resampling technique used to assess machine learning models on a small data sample, in order to assure robustness and reliability. The decision function for SVM with RBF kernel can be represented as: With an RBF kernel, the SVM decision function is expressed as:


$$ \mathrm{f}(x)=\sum_{i=1}^n\alpha iyiK\left(x, xi\right)+b $$

In this case, the Lagrange multipliers are represented by αi, the class labels by $yi,$ the kernel function by K, the bias term by b, and the support vectors by xi.

The projected outcome for RF may be shown as:


$$ \hat{y} = mode\left(y1,y2,\dots, yn\right) $$

where the individual tree predictions are denoted by *y*1, *y*2, ..., *yn* and the predicted class is represented by $ \hat{y}$

One way to describe the DT decision rule is as:


$$ if\,X\,feature \le thresholdthenclass= leftchildelseclass= rightchild $$

Given the GNB features, the likelihood of a class may be expressed as follows:


$$ P\left(y|x1,x2,\dots, xn\right)=\frac{=P(y){\prod}_{i=1}^nP\left( xi|y\right)}{P\left(x1,x2,\dots, xn\right)} $$

The XGBoost forecast can be shown as:


$$ \hat{y} =\sum_{m=1}^M fm(x) $$

where the forecast of the $m-th$ tree is denoted by $fm(x)$ and *M* is the number of trees.

## Results

### Identification of differentially expressed genes

**Figure 2:**
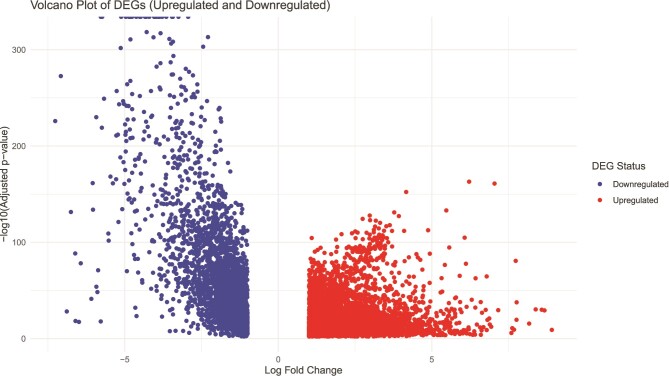
Volcano plot of DEGs. The DEGs are obtained based on criteria of log fold-change (LogFC) <1 for downregulated genes and (IogFC) > 1 for upregulated genes with a *P*-value < 0.05.

Seven thousand six hundred thirty-seven genes were ultimately found to be differentially expressed when breast cancer tissues were compared to normal tissues for differential expression analysis. Figure 2 depicts the volcano plot of differential expression results where red dots represent upregulated DEGs and blue dots represent downregulated DEGs. Table 1 describes the top 10 upregulated and top 10 downregulated DEGs of the BRCA dataset.

**Table 1:** Top 10 upregulated and top 10 downregulated DEGs of BRCA.

Gene symbol	Description	Regulation
UCN3	Urocortin-3	Upregulated
MUC2	Mucin 2	Upregulated
CGA	Glycoprotein hormones, alpha polypeptide	Upregulated
CSAG1	Chondrosarcoma-associated gene	Upregulated
MAGEA12	MAGE family member A12	Upregulated
ACTL8	Actin like 8	Upregulated
MAGEA1	MAGE family member A1	Upregulated
IBSP	Integrin binding sialoprotein	Upregulated
KLHL1	Kelch-like family member 1	Upregulated
MAGEA3	MAGE family member A3	Upregulated
MYH2	Myosin heavy chain 2	Downregulated
CKM	Creatine kinase	Downregulated
MIR1–1HG	MIR1–1 host gene	Downregulated
MYL2	Myosin Light Chain 2	Downregulated
MYH7	Myosin Heavy Chain 7	Downregulated
PPP1R3A	Protein Phosphatase 1 Regulatory Subunit 3A	Downregulated
MYL1	Myosin Light Chain 1	Downregulated
STRIT1	Small Transmembrane Regulator of Ion Transport 1	Downregulated
C10orf71	Chromosome 10 Open Reading Frame 71 Protein Coding Gene	Downregulated
NRAP	Nebulin-related anchoring protein	Downregulated

### Analysis of protein–protein interaction

**Figure 3:**
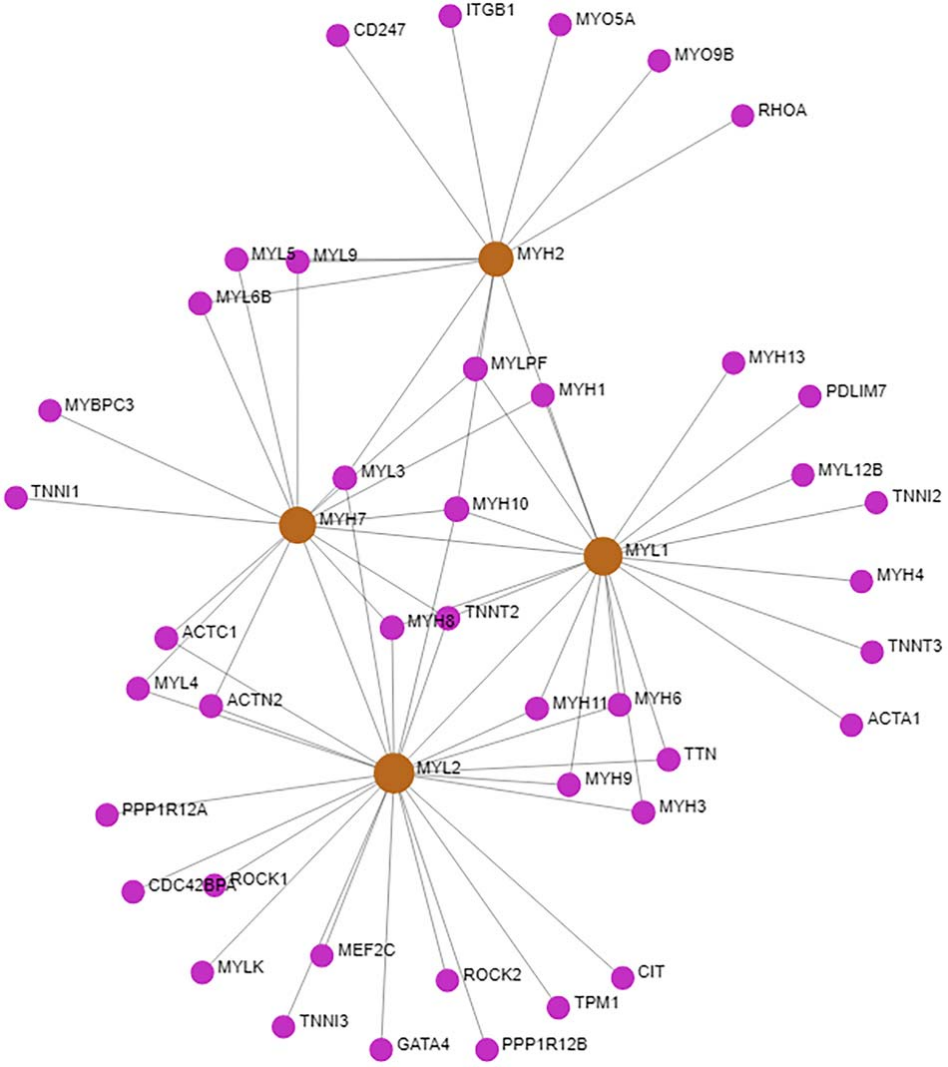
The PPI network of the top 10 upregulated and top 10 downregulated genes of BRCA. The bigger circle with different colors represents the top 4 hub proteins.

In order to obtain hub-bottleneck genes, we have generated a PPI network using the top 10 upregulated and top 10 downregulated genes from the DEGs. Figure 3 represents the PPI network of the top 10 upregulated and top 10 downregulated genes of BRCA. We have identified four hub-bottleneck genes, i.e. MYH2, MYL1, MYL2, and MYH7 from the PPI analysis.

### Identification of signaling and gene ontology terms

We employed a gene set enrichment analysis to obtain ontological and signaling pathways. In this analysis, we used the top 10 upregulated and top 10 downregulated genes from the obtained DEGs to identify signaling pathways using five global pathway databases, including KEGG, BioPlanet, and BioCarta. Molecular function, biological process, and cellular component were the three classifications from which the ontological pathways were obtained. The top 10 signaling and ontological pathways based on the adj *P*-value are represented in Tables 2 and 3, respectively.

**Table 2:** The top 10 signaling pathways of the DEGs obtained from BRCA.

Category	Pathway	Genes in the pathway	*P*-value
KEGG	Cardiac muscle contraction	MYL2,MYH7	.003377564
	Hypertrophic cardiomyopathy	MYL2, MYH7	.003609434
	Dilated cardiomyopathy MYL2,MYH7 0.004094897	MYL2,MYH7	.004094897
	Adrenergic signaling in cardiomyocytes	MYL2,MYH7	.009716646
	Focal adhesion	IBSP,MYL2	.016953582
	Neuroactive ligand–receptor interaction	UCN3,CGA	.044995524
	Arginine and proline metabolism	CKM	.04885255
	Ovarian steroidogenesis	CGA	.049806079
	Autoimmune thyroid disease	CGA	.051710413
	Regulation of lipolysis in adipocytes	CGA	.053611122
BioPlanet	Tight junction	MYH2,MYL2,MYH7	3.02E-04
	Striated muscle contraction	MYL1,MYL2	6.54E-04
	Muscle contraction	MYL1,MYL2	.001086179
	Retinoblastoma protein regulation	CKM,MYL1	.001961104
	NFAT involvement in hypertrophy of the heart	MYH2,MYL2	.002141024
	Viral myocarditis	MYH2,MYH7	.002265166
	Cardiac muscle contraction	MYL2,MYH7	.00286498
	Dilated cardiomyopathy	MYL2,MYH7	.0044345
	Glycoprotein hormones	CGA	.004990441
	SARS coronavirus protease	CKM	.006979996
BioCarta	Regulators of Bone Mineralization *Homo sapiens* h npp1Pathway	IBSP	.01094778
	PKC-catalyzed phosphorylation of inhibitory phosphoprotein of myosin phosphatase *H. sapiens* h myosinPathway M	MYL2	.020801468
	CCR3 signaling in Eosinophils *H. sapiens* h CCR3Pathway	MYL2	.022760983
	ALK in cardiac myocytes *H. sapiens* h alkPathway	MYL2	.026668846
	NFkB activation by nontypeable Hemophilus influenzae *H. sapiens* h nthiPathway	MYL2	.028617205
	Rho cell motility signaling pathway *H. sapiens* h rhoPathway	MYL2	.0315328
	Rac 1 cell motility signaling pathway *H. sapiens* h rac1Pathway	MYL2	.03540733
	Trefoil factors initiate mucosal healing *H. sapiens* h tffPathway	MUC2	.03540733
	NFAT and hypertrophy of the heart *H. sapiens* h nfatPathway	MYL2	.043112259

**Table 3:** The top 10 gene ontology terms of the DEGs obtained from BRCA.

Category	Pathway	Genes in the pathway	*P*-value
Molecular function	Histone deacetylase binding	MAGEA12, MAGEA1, MAGEA3	1.26E-04
	Actin binding	MYL2, NRAP, KLHL1	7.66E-04
	Myosin heavy chain binding	MYL2	.005985691
	Cuprous ion binding	MUC2	.007973357
	Caspase binding	MAGEA3	.012926021
	Peptide hormone receptor binding	UCN3	.012926021
	Muscle alpha-actinin binding	NRAP	.013913732
	Neuropeptide receptor binding	UCN3	.016871235
	Protein phosphatase 1 binding	PPP1R3A	.016871235
	Actin monomer binding	MYL2	.022760983
	Alpha-actinin binding	NRAP	.022760983
	Copper ion binding	MUC2	.044071254
	Myosin binding	MYL2	.05266122
	Hormone activity	CGA	.071488248
Biological process	Actin-myosin filament sliding	MYH2, MYL1, MYH7	4.25E-05
	Muscle filament sliding	MYL1, MYH7	9.90E-05
	Cardiac myofibril assembly	MYL2, NRAP	1.08E-04
	Muscle contraction	MYH2, MYL1, MYH7	2.81E-04
	Ventricular cardiac muscle tissue development	MYL2, MYH7	4.34E-04
	Cardiac muscle tissue morphogenesis	MYL2, MYH7	4.92E-04
	Cardiac muscle contraction	MYL2, MYH7	4.92E-04
	Ventricular cardiac muscle tissue morphogenesis	MYL2, MYH7	4.92E-04
	Positive regulation of intracellular transport	MYL1, STRIT1	6.89E-04
	Heart contraction	MYL2, MYH7	7.61E-04
	Cardiac ventricle morphogenesis	MYL2, MYH7	7.99E-04
	Myofibril assembly	MYL2, MYH7	9.58E-04
	Striated muscle contraction	MYL2, MYH7	.001467055
	Host-mediated regulation of intestinal microbiota composition	MUC2	.005985691
Cellular component	Myofibril	MYH2, MYL1, MYL2, MYH7	1.70E-08
	Muscle myosin complex	MYH2, MYL1, MYH7	3.09E-07
	Myosin filament	MYH2, MYH7	9.90E-05
	Supramolecular fiber	MYH2, MYH7	5.86E-04
	Golgi lumen	MUC2, CGA	.0044345
	Sarcoplasmic reticulum membrane	STRIT1	.026668846
	Intercalated disc	NRAP	.03056186
	Cell–cell junction	MYH2, NRAP	.035451148
	Actin cytoskeleton	MYL2, ACTL8	.04171588
	Sarcoplasmic reticulum	STRIT1	.044071254
	Caveola	MYL1	.06023514
	Actin filament	ACTL8	.068687033

### Identification of differentially expressed genes–microRNA and transcription factor–gene interaction

**Figure 4:**
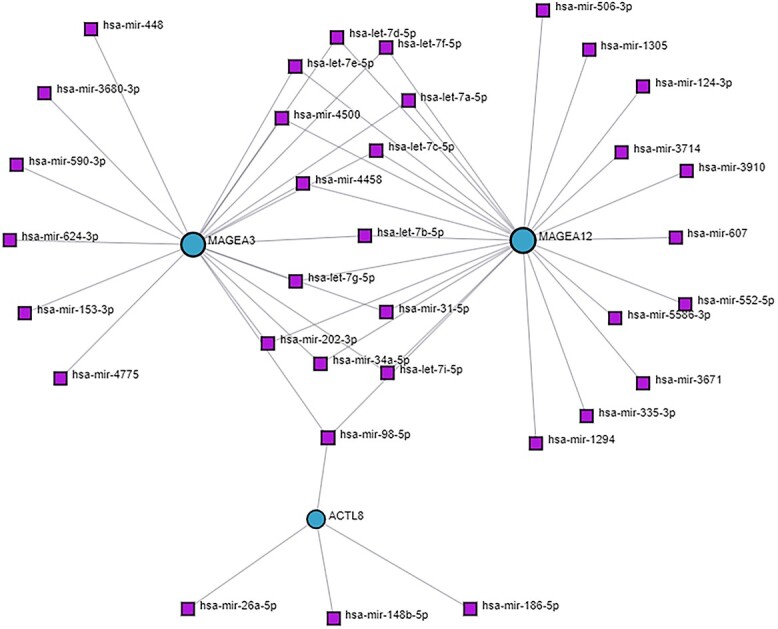
miRNA–gene interaction regulatory network. Target regulatory molecules are represented by square nodes, while associated genes are represented by circular nodes.

With the top 10 up- and downregulated DEGs from BRCA, we were able to obtain regulatory components from miRNA–DEGs and TF–DEGs interactions. Figure 4 represents the miRNA–DEGs interactions. In Fig. 4, purple squares represent the miRNAs, and sky-blue circles represent the DEGs.

**Figure 5:**
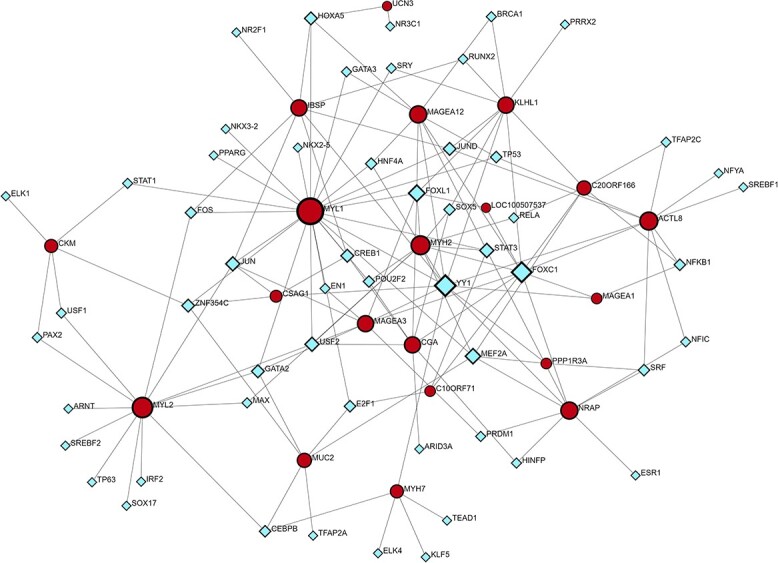
TF–gene interaction regulatory network. Square nodes indicate target regulatory molecules (TFs), and circular shape nodes represent the associated DEGs.

Figure 5 represents the TF–DEGs interactions based on the top 10 up- and downregulated genes. In Fig. 5, indigo-blue rhombus shape nodes represent the TFs, and the red circular shape nodes represent the associated DEGs. Based on the degree of a node, its dimension is generated. Four red circular nodes, namely, MYL1, MYH2, MYL2, and ACTL8, are considered as significant hub genes, and four rhombus TFs, namely, YY1, FOXC1, FOXL1, and MEF2A, are considered as regulatory molecules.

### Identification of protein–drug and protein–chemical interactions

**Figure 6:**
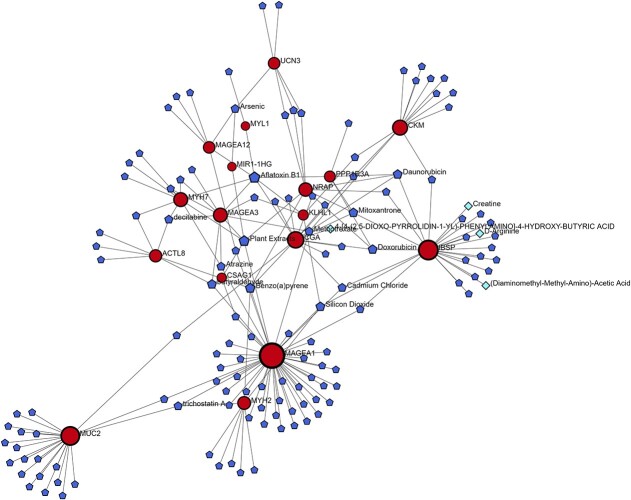
Combined protein–drug and protein–chemical interaction network. Pentangle nodes indicate chemical compounds and rhombus nodes indicate drug regulatory molecules.

Figure 6 represents the combined protein–drug and protein–chemical network based on the top 10 up- and downregulated genes obtained from BRCA. In Fig. 6, the red circular nodes indicate drugs, and the blue pentangle nodes represent the chemical compounds that have an impact on how genes are expressed. The proteins MAGFA1, MUC2, IBSP, CKM, and MYH2 were considered as the highly expressed therapeutic targets in the combined network.

### Survival analysis results

**Figure 7:**
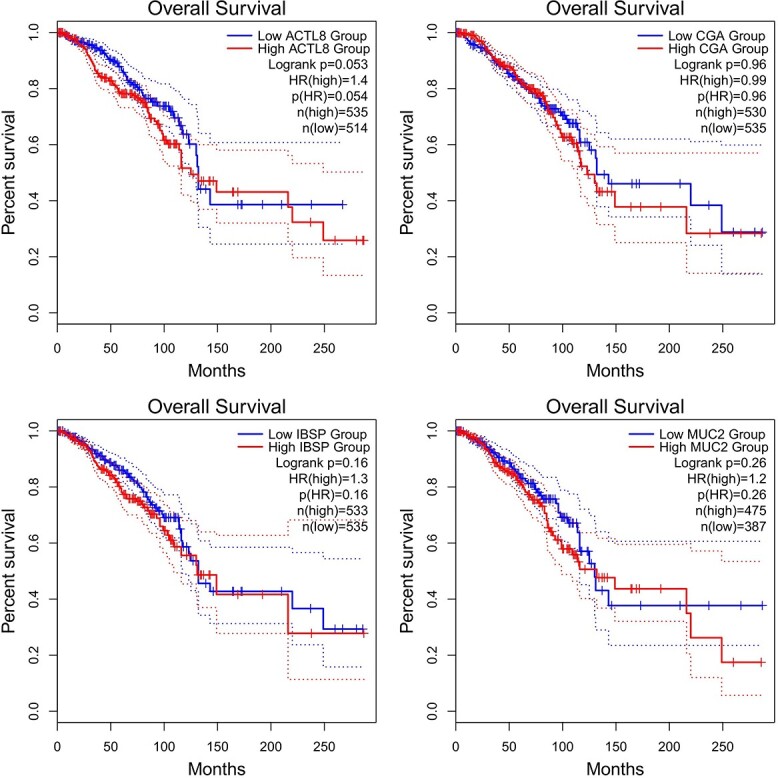
Overall survival rate of the genes ACTL8, CGA, IBSP, and MUC2.

Survival analysis revealed that only 4 genes among the top 10 upregulated genes and downregulated genes found from DEG analysis were associated with the prognosis of breast cancer, namely, ACTL8, CGA, IBSP, and MUC2 genes, and their survival curves are shown in Fig. 7.

### Results of different machine learning models

Table 4 shows the assessment metrics for several machine learning models used to classify different stages of breast cancer. Each model was evaluated using numerous performance indicators, including accuracy, precision, recall, F1 score, and specificity. The stages of breast cancer were divided into three categories: I–II, III, IV, V; I, II–III, IV, V; and I, II, III–IV, V, allowing for a thorough examination across stages. Notably, RF and XGBoost consistently achieved good accuracy, precision, recall, and F1 scores across different stages of breast cancer. These models’ accuracy evaluations, which indicate their capacity to distinguish between different cancer stages, varied from 85.51% to 97.19% for RF and from 85.51% to 95.23% for XGBoost. Excellent accuracy ratings were generated by both RF and XGBoost, ranging from 85.58% to 97.20% for RF and from 85.59% to 95.34% for XGBoost. These results show that both methods can consistently recognize real positive circumstances. The high-recall figures (85.51%–97.19% for RF and 85.51%–95.23% for XGBoost) show that both techniques were successful in gathering all positive examples. Moreover, RF and XGBoost routinely had high F1 ratings, which show the harmonic mean of accuracy and recall, indicating their overall efficacy. With an accuracy range of 63.30%–85.19%, the SVM also performed well, most notably in differentiating between phases I–II and III, IV, and V.

**Table 4:** Model evaluation metrics.

Stage	Model	Accuracy	Precision	Recall	F1	Specificity
I–II, III, IV, V	RF	94.46%	94.61%	94.46%	94.45%	93.77%
	SVM	85.19%	84.19%	85.19%	84.98%	77.16%
	DT	83.66%	83.44%	84.66%	83.47%	67.50%
	GaussianNB	79.76%	68.02%	73.33%	69.42%	36.23%
	KNN	80.63%	68.02%	73.33%	69.42%	60.89%
	XGBoost	93.01%	93.27%	93.01%	92.99%	92.38%
I, II–III, IV, V	RF	97.19%	97.20%	97.19%	97.18%	92.88%
	SVM	77.95%	77.95%	77.95%	76.56%	83.90%
	DT	85.19%	84.19%	85.19%	84.98%	77.16%
	GaussianNB	83.74%	86.25%	83.74%	82.65%	97.36%
	KNN	77.77%	80.23%	77.70%	73.46%	96.08%
	XGBoost	95.23%	95.34%	95.23%	95.23%	87.45%
I, II, III–IV, V	RF	85.51%	85.58%	85.51%	85.53%	89.71%
	SVM	63.30%	63.43%	63.30%	61.87%	82.69%
	DT	67.22%	67.00%	67.22%	67.00%	74.44%
	GaussianNB	74.82%	77.85%	74.82%	72.67%	96.13%
	XGBoost	85.51%	85.84%	85.51%	85.59%	88.78%

However, compared to RF and XGBoost, the SVM has a lower specificity, suggesting that it would have trouble correctly identifying actual negative scenarios. Phase-by-phase variations in the DT’s accuracy ranged from 67.22% to 85.19%. While the DT had poorer accuracy than RF and XGBoost, it nevertheless obtained acceptable performance metrics, demonstrating its potential value in specific applications. GNB showed lower accuracy than other models, ranging from 63.30% to 83.74%. This shows that GNB may be less efficient at reflecting the complexity of breast cancer staging than more advanced models such as RF and XGBoost. KNNs achieved reasonable accuracy, ranging from 77.77% to 80.63%. While the KNN demonstrated lesser accuracy than RF and XGBoost, its performance was nonetheless respectable, demonstrating its potential use in certain scenarios.

**Figure 8:**
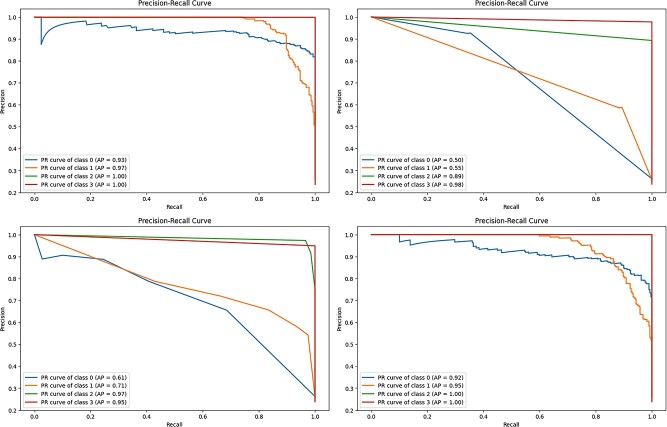
Precision–recall curve of machine learning models RF, GNB, KNNs, and XGB (XGBoost).

**Figure 9:**
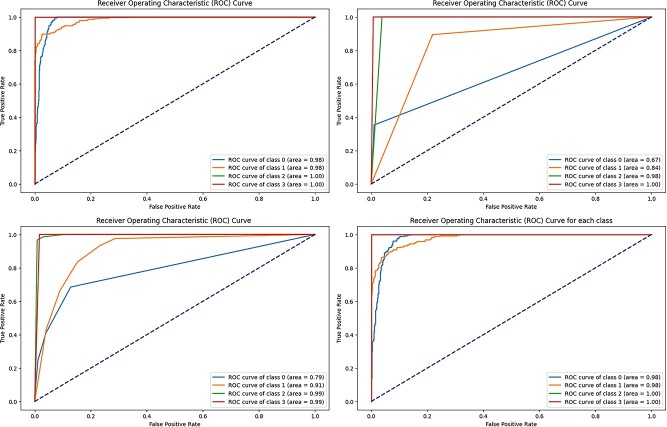
ROC curve of machine learning models RF, GNB, KNN, and XGB.

Overall, the results show that RF and XGBoost are successful in properly classifying different stages of breast cancer, implying that they might be used in clinical practice for precise diagnosis and treatment planning. Figure 8 displays the precision–recall curve, while Fig. 9 displays the ROC curve for the four distinct models.

## Discussion

In women globally, breast cancer is the primary cause of cancer-related death. In both developed and developing nations, it comes in second place among the main causes of cancer-related deaths. Treatment effectiveness dramatically reduces metastasis and postcarcinogenesis, highlighting how crucial early identification is. Not only does prompt diagnosis increase patient survival rates, but it also makes it easier to put therapies into place that can reduce morbidity and increase overall survival rates [45]. Breast cancer screening in various European countries is mostly performed via mammography or breast magnetic resonance imaging (MRI), although these technologies present problems. Although breast MRI can be costly and has certain drawbacks, radiologists’ experience is crucial in interpreting mammograms. Both screening techniques are widely used; however, it is important to carefully weigh their advantages and disadvantages [46]. In this study, we focus on identifying and classifying breast cancer using system biology and machine learning approaches. We have studied the breast cancer gene expression data from TCGA datasets. First of all, we utilized the DEGs of the gene expression data of TCGA datasets, and then, we found 20 DEGs including 10 upregulated and 10 downregulated genes of breast cancer (Table 1). To discover prospective therapeutic targets, we examined differentially expressed breast cancer genes using PPI, molecular signaling pathway, ontology, and regulatory analysis. Similarly, system biology studies were done to study the molecular markers and therapeutic targets by Barua *et al*. [47–49]. We utilized gene enrichment analysis to obtain the responsible genes of breast cancer by discovering gene ontologies and pathways. We identified 20 DEGs using gene ontological exploration based on *P*-value to obtain insight into the molecular importance of breast cancer. The three types of GO analysis such as molecular function (molecular-level performance), biological process (biological activities), and cellular component (gene regulatory activities) were employed from the GO database using Enrichr as an annotation source [50]. In the molecular function, histone deacetylase binding and actin binding activity are significant among the top GO terms. Actin-myosin filament sliding and muscle filament sliding for biological processes and myofibril and muscle myosin complex for cellular components are among the top GO terms.

Myofibrils are the complex structures found inside muscle cells. They are essential for enabling muscular contraction, which is a basic mechanism that is necessary for movement and body function. This process also greatly contributes to general physiological balance. Several muscle diseases and ailments that impair movement and physical well-being may be related to myofibril dysfunction or dysregulation [51]. Histone deacetylases (HDACs) in breast cancer regulate gene expression by altering histone proteins. This can affect the expression of genes involved in cell growth, metastasis, and medication resistance [52]. HDAC inhibitors have shown promise as possible therapies because they reverse these processes and make cancer cells more susceptible to therapy. Another study showed that alterations in cytoskeletal dynamics, including gactin–myosin interactions, can indirectly influence various aspects of breast cancer progression [53, 54]. Enrichment analysis is an important tool for identifying correlations unique to breast cancer and other disorders [55, 56]. The KEGG pathway of the DEGs revealed the top four pathways: cardiac muscle contraction, hypertrophic cardiomyopathy, dilated cardiomyopathy, and adrenergic signaling in cardiomyocytes. In a study, cardiac muscle contraction pathways could potentially impact breast cancer growth by common molecular processes or systemic consequences, as shown by the linked signaling networks in cancer-associated fibroblasts and the tumor microenvironment [57]. According to some research, there seems to be a link between the advancement of breast cancer and chronic stress, which can trigger adrenergic signaling pathways [58]. In addition, Tight junction and Striated muscle contraction for BioPlanet pathway, and Regulators of Bone Mineralization *Homo sapiens*h npp1Pathway and PKC-catalyzed phosphorylation of inhibitory phosphoprotein of myosin phosphatase *H. sapiens* h myosinPathway for BioCarta pathway were revealed as the top significant pathways (Table 2). It has been found that frequent exercise, which burns calories through muscular contraction and may affect the metabolism of creatine, lowers the risk of breast cancer [59, 60]. Moreover, abnormal metabolism including the metabolism of creatine may contribute to the advancement of cancer [61, 62]. The Striated Muscle Contraction Pathway, PtdIns 4 5 P2 In Cytokinesis Pathway, and Osteoblast Signaling are all thought to have a role in breast cancer. While these pathways are largely concerned with muscle function, cell division, and bone growth, there may be indirect links or common regulatory mechanisms with breast cancer. Dysregulation of cell division signaling pathways, such as cytokinesis, has been linked to the development of cancer. Furthermore, modifications in bone signaling pathways may impact the bone microenvironment, influencing the course of breast cancer bone metastases [63, 64]. Analysis of protein–protein networks is a crucial method for determining the processes behind the development of illness [65, 66]. In order to acquire hub proteins, we built a network of interactions between proteins. The PPI analysis revealed four hub proteins that are MYH2, MYL1, MYL2, and MYH7. It’s worth noting that changes in numerous cytoskeleton components, such as myosin and myosin-associated proteins, have been linked to cancer development, particularly breast cancer. These modifications can impact cell motility, invasion, and metastasis, which are important factors in cancer growth [67, 68]. miRNAs and TFs regulate gene expression through post-transcriptional and transcriptional mechanisms. The dysregulation of miRNAs and TFs has emerged as a critical mechanism in breast cancer pathogenesis, impacting multiple aspects of tumor initiation, development, and metastasis. Several studies have highlighted the deregulation of certain microRNAs in breast cancer, such as miR-21, miR-155, and miR-221, which are typically overexpressed and linked with poor prognosis [69, 70]. By specifically targeting oncogenes or important tumor suppressor genes, these miRNAs can alter vital signaling pathways that are involved in invasion, apoptosis, and cell proliferation. Furthermore, abnormal expression of TFs, including members of the E2F, FOX, and AP-1 families, has been linked to breast cancer development [71, 72]. TFs control the expression of genes involved in a variety of biological functions. When TFs are dysregulated, normal gene expression patterns can be disrupted, which can contribute to the development of cancer. We identified the top significant miRNA targets (hsa-let-7d-5p, hsa-mir-4500, hsa-mir-34a-5p, hsa-let-7a-5p, and hsalet-7c-5p) which may be interconnected with pathways of breast cancer (Fig. 3). The target miRNAs may be regarded biomarkers and therapeutic targets to treat breast cancer [73, 74]. The top significant regulatory TFs (YY1, FOXC1, FOXL1, and MEF2A) may be responsible for the related pathways of the breast cancer cellular process of disease development. Among the discovered TFs, YY1 has been implicated in tumor aggressiveness and medication resistance through the regulation of cell cycle control and metastasis-related genes [75]. FOXC1, on the other hand, promotes tumor growth and metastasis by regulating genes associated with epithelial–mesenchymal transition and angiogenesis, contributing to poor clinical outcomes [76]. In another study [77], researchers discovered that while the specific role of FOXL1 in breast cancer remains unclear, accumulating evidence shows that it may have tumor-suppressive activities, reducing proliferation and invasion in breast cancer cells. Meanwhile, MEF2A has been linked to boosting tumor development and metastasis via modulating genes involved in cell proliferation and survival, indicating a bad prognosis in breast cancer patients [78].

Apart from clarifying the functions of TFs in breast cancer, protein–chemical interaction research has discovered other possible targets for treatment. Because it inhibits dihydrofolate reductase and messes with DNA synthesis, methotrexate, a commonly used chemotherapeutic treatment, has demonstrated success in treating a variety of malignancies, including breast cancer [79]. Because of its capacity to cause DNA damage and encourage carcinogenesis, benzopyrene, a polycyclic aromatic hydrocarbon present in tobacco smoke, has been linked to the development of breast cancer [80]. 4-[4-(2,5-Dioxo-pyrroldin-1-yl)-phenylamino] is the compound. Despite not having been well researched, -4-hydroxy-butyric acid shows promise as a therapeutic agent since it targets particular biochemical pathways that are implicated in the advancement of breast cancer [81].

Furthermore, machine learning analysis is implemented to improve the accuracy of breast cancer stage classifications. Notably, RF and XGBoost consistently delivered excellent accuracy, precision, recall, and F1 scores throughout all stages, with RF ranging from 85.51% to 97.20% and XGBoost from 85.51% to 95.34%. The SVM was effective in distinguishing between phases I–II and III, IV, and V, with accuracy ranging from 63.30% to 85.19%. However, the SVM had poorer specificity than RF and XGBoost. DT performance varied, although GNB accuracy was lower. KNNs demonstrated reasonable accuracy. Overall, RF and XGBoost indicate potential for therapeutic usage in precise breast cancer staging.

This research has several benefits for the identification, categorization, and staging of breast cancer. The TCGA dataset combines bioinformatics and machine learning to offer a thorough examination of molecular markers and cancer development. High diagnosis accuracy (97.19% and 95.23%, respectively) is obtained by the application of machine learning models like RF and XGBoost, which may be very helpful for clinical practice. More focused treatment approaches are also made possible by the discovery of possible therapeutic targets through the examination of signaling cascades, PPIs, and DEGs. Finding important proteins and miRNAs connected to the development of cancer also provides useful biomarkers for early identification and individualized treatment. Through the implementation of systems biology methods, the research expands our knowledge of the molecular pathways underlying breast cancer and helps to advance personalized medicine strategies that customize treatment regimens based on the unique characteristics of each patient. All things considered, this research offers a strong foundation for improving breast cancer detection, staging, and treatment advancement. The code of the project is available at the following link: https://github.com/dassaurav404/Breast-Cancer-Classification-using-Machine-Learning-andBioinformatics-Approach.git.

## Conclusions

The complete investigation of breast cancer utilizing systems biology and machine learning methodologies has revealed important information about disease processes and prospective treatment targets. Significant molecular activities such as histone deacetylase binding and actin binding were found among the elevated genes, suggesting that they have active roles in cancer development. Similarly, downregulated genes were linked to key biological processes such as muscle filament sliding, indicating a possible imbalance in cellular functions. Enrichment analysis helped to understand the pathways that stimulate breast cancer development. The main pathways identified, including heart muscle contraction and adrenergic signaling, indicate possible linkages between cancer and systemic processes such as chronic stress. Furthermore, the enrichment of pathways associated with muscle contraction and cytokinesis emphasizes the role of cytoskeletal dynamics in cancer development, opening up new possibilities for therapeutic intervention. The PPI study revealed hub proteins such as MYH2 and MYH7, demonstrating the role of cytoskeletal components in breast cancer etiology. MicroRNA and TF studies revealed dysregulation of key regulators such as hsa-let-7d-5p and YY1, indicating their potential as diagnostic indicators and therapeutic targets. Furthermore, the discovery of chemicals with therapeutic potential, such as methotrexate and 4-hydroxy-butyric acid, emphasizes the need to address particular biochemical pathways in cancer treatment. In addition, machine learning studies showed that models such as RF and XGBoost can reliably detect breast cancer stages, with RF attaining an accuracy range of 85.51%–97.20% and XGBoost ranging from 85.51% to 95.34%. The SVM has shown success in differentiating between phases I–II and III, IV, and V, with an accuracy range of 63.30%–85.19%. However, the SVM has lower specificity than RF and XGBoost. One key limitation of the study is the lack of clinical trials to validate the findings. While the machine learning models show high accuracy in cancer classification, their clinical applicability requires further testing to confirm their effectiveness in real-world settings. Additionally, the study primarily focuses on specific subtypes and stages of breast cancer, necessitating further research to assess the methodology’s effectiveness across a broader range of breast cancer types and diverse patient populations. Another key limitation of this study is the lack of clinical trials to validate the findings, as the machine learning models, while demonstrating high accuracy in classification, require further testing to assess their clinical applicability. Additionally, the study’s focus on specific subtypes and stages of breast cancer limits its scope, and further research is needed to evaluate the methodology’s effectiveness across a wider range of breast cancer types and diverse patient populations. Despite the use of SMOTE to address class imbalance, the dataset’s imbalance, particularly in later-stage cancers, may still impact model performance and its ability to generalize effectively. The study’s research findings can be used in laboratory studies to better understand potential therapeutic targets for breast cancer treatments.

**Key Points**

- Determine the genes responsible for breast cancer and construct a protein–protein interaction (PPI) network.
- Predict candidate drugs based on the PPI network’s hub nodes and identify the diseases associated with the hub genes.
- Role of biomarkers.
- Cross-validate the expression level of the hub genes and perform survival analysis.
- Application of machine learning in cancer diagnosis.

## Supplementary Material

Comprehensive_Bioinformatics_and_Machine_Learning_Analysis_bbae628

## Data Availability

The datasets for this study were collected from the Genomic Data Commons (GDC) Data Portal (https://portal.gdc.cancer.gov/repository), a publicly available repository for cancer analysis.
