# Supplementary material for: Comprehensive bioinformatics and machine learning analyses for breast cancer staging using TCGA dataset
Source: Brief Bioinform. 2024 Dec 4;26(1):bbae628. doi: 10.1093/bib/bbae628 (PMC11630003; doi:10.1093/bib/bbae628)
Supplement: Comprehensive_Bioinformatics_and_Machine_Learning_Analysis_bbae628 [file comprehensive_bioinformatics_and_machine_learning_analysis_bbae628.docx]

Comprehensive Bioinformatics and Machine Learning Analyses for Breast Cancer Staging Using TCGA Dataset

Saurav Chandra Das^a,c^, Wahia Tasnim^b^, Humayan Kabir Rana^b^, Uzzal Kumar Acharjee^a,^,

Md. Monowarul Islam^a^, Rabea Khatun^b^

*^a^Department of Computer Science and Engineering, Jagannath University, Dhaka, Bangladesh*

*^b^Department of Computer Science and Engineering, Green University of Bangladesh, Narayanganj-1461,*

*Dhaka, Bangladesh*

*^c^ Department of Internet of Things and Robotics Engineering, Bangabandhu Sheikh Mujibur Rahman Digital University, Bangladesh*

# Abstract

**Background:** Breast cancer is an alarming global health concern, including a vast and varied set of illnesses with different molecular characteristics. The fusion of sophisticated computational methodologies with extensive biological datasets has emerged as an effective strategy for unravelling complex patterns in cancer oncology.

**Methods:**This research delves into breast cancer staging, classification, and diagnosis by leveraging the comprehensive dataset provided by the TCGA. By integrating advanced ma- chine learning algorithms with bioinformatics analysis, it introduces a cutting-edge method- ology for identifying complex molecular signatures associated with different subtypes and stages of breast cancer.

**Results:** This study utilizes TCGA gene expression data to detect and categorize breast cancer through the application of machine learning and systems biology techniques. Re- searchers identified differentially expressed genes (DEGs) in breast cancer and analyzed them using signaling pathways, protein-protein interactions, and regulatory networks to un- cover potential therapeutic targets. The study also highlights the roles of specific proteins (MYH2, MYL1, MYL2, MYH7) and microRNAs (such as hsa-let-7d-5p) which are the po- tential biomarkers in cancer progression founded on several analyses. In terms of diagnostic accuracy for cancer staging, the Random Forest method achieved 97.19%, while the XG- Boost algorithm attained 95.23%.

**Conclusions:** Bioinformatics and machine learning meet in this study to find potential biomarkers that influence the progression of breast cancer. The combination of sophis- ticated analytical methods and extensive genomic datasets presents a promising path for expanding our understanding and enhancing clinical outcomes in identifying and cate- gorising this intricate illness. The code of the project is available at the following link: https://github.com/dassaurav404/Breast-Cancer-Classification-using-Machine-Learning-and- Bioinformatics-Approach.git

*Keywords:* Breast Cancer; TCGA; Cancer Staging; Ontology; Machine Learning; Transcription Factors

1

# Introduction

Breast cancer is the second most common cancer among women, following skin cancer, and is the second leading cause of cancer-related mortality, after lung cancer [[1](#_bookmark12)]. Globally, breast cancer has surpassed lung cancer as the most frequently diagnosed cancer in women. In 2020, an estimated 2,261,419 women worldwide were diagnosed with breast cancer [[2](#_bookmark13)]. According to the American Society for Clinical Oncology, it is projected that in 2023, 297,790 women in the United States will be diagnosed with invasive breast cancer, while 55,720 will be diagnosed with non-invasive (in situ) breast cancer. Since the mid-2000s, the incidence of invasive breast cancer in women has increased by approximately 0.5% annually, likely driven by factors such as declining fertility rates, delayed age of first childbirth, and rising obesity rates. Additionally, invasive breast cancer is expected to affect around 2,800 men in the U.S. in 2023 [[3](#_bookmark14)]. Early detection of breast cancer is critical for selecting appropriate treatments and reducing the risk of metastasis [[4](#_bookmark15)]. Breast cancer is a heterogeneous and evolving disease, marked by various somatic mutations and changes in gene and protein expression. It is classified into several subtypes based on the expression of the progesterone receptor (PR), estrogen receptor (ER), and human epidermal growth factor receptor 2 (HER2). Each subtype requires specific treatment approaches, which can affect drug resistance, cancer recurrence, and mortality rates [[5](#_bookmark16), [6](#_bookmark17)]. Identifying novel clinical biomarkers is essential for better patient stratification, enhancing the accuracy of initial diagnoses, and monitoring the progression, metastasis, and recurrence of breast cancer [[7](#_bookmark18)].

In present days, tumour markers have become increasingly prevalent in areas of cancer

detection and therapy. For tumour screening, diagnosis, efficacy and prognosis evaluation, recurrence detection, and so forth, the optimal tumour marker should possess high specificity, possess the ability to recognize tiny lesions, and quantify the tumour burden [[8](#_bookmark19)]. Staging a cancer is the process of quantifying the extent of the cancer’s metastasis throughout the body. The process of measuring and evaluating the extent to which cancer has progressed to various sections of the body is referred to as cancer staging. It is helpful in choosing the most efficient kind of therapy as well as detecting the degree to which the cancer has spread. Additionally, it is used by physicians in the process of calculating survival rates. The Joint Working Committee for Cancer Tumor-Lymph Node-Metastasis (TNM) states that there are typically five different stages of cancer: Stages 0, I, II, III, and IV [[9](#_bookmark20)].In addition to determining the cancer’s size and location, the stage of the disease will also impact the existence of indicators of cancer spread and how much cancer has progressed to neighbouring tissues, lymph nodes, and other body regions [[10](#_bookmark21)]. For individuals between the ages of 18 and 55 who suffer from breast cancer, with the greatest grade detected at stage I, the five-year survival rate is 97% and can be cured with the right care, whereas the projected five-year survival rates of stages II, III, and IV are 92%, 77%, and 28%, respectively [[11](#_bookmark22)]. Among all cancers, breast cancer mutations are the most prevalent and lethal. A patient’s chances of survival are significantly increased when the illness is identified in its early stages [[12](#_bookmark23)].

*Preprint submitted to Briefings in Bioinformatics November 14, 2024*

Machine learning could uncover correlations that are difficult to recognize in vast, noisy, or complicated datasets. This skill is specifically appropriate for data analysis applica- tions in the healthcare sector, particularly those requiring intricate proteomics and genomic expression-based applications, which have been employed commonly in recent years for the identification and treatment of cancer[[13](#_bookmark24)]. In the medical area, machine learning techniques are commonly employed including random forests [[14](#_bookmark25)], SVM [[15](#_bookmark26), [16](#_bookmark27), [17](#_bookmark28)] and decision trees [[18](#_bookmark29), [19](#_bookmark30)]. Applications like Xie et al.’s [[20](#_bookmark31)] employed spectral data to create SVM models with an average accuracy of 100% for quick and noninvasive keratitis detection, as well as SVM and DT models were utilized by Chen Fangfang et al.[[21](#_bookmark32)] to quickly detect gliomas with a predictive accuracy of about 90%. It demonstrates even more that machine learning is more applicable to the diagnosis of diseases. A supervised learning model called SVM is capable of handling both linear and nonlinear problems. It works to address issues with classification and regression. The ultimate class of a test object is determined by combining a collection of decision trees that are randomly chosen from the training set. Random forests are a powerful force in machine learning because decision trees, which are renowned for their adaptability, excel in classification and regression tasks.

In the field of biomedicine, there have been some encouraging results using gene network-

based cancer prediction and biomarker screening. Sheikh Jubair et al.’s[[22](#_bookmark33)] subtype-specific network biomarker approach, for example, has demonstrated high predictive effectiveness for identifying the survivorship of breast cancer patients with it. Shiyan Li et al.[[23](#_bookmark34)] constructed a model to predict the prognosis of cervical cancer patients using the weighted gene co- expression network (WGCNA) paired alongside the LASSO technique and showed that the approach is legitimate and reliable. In this work, we first performed a differential expression analysis between breast cancer and healthy controls. The LASSO feature selection approach has been employed in a variety of biological applications. Lasso is a well-known feature selection approach that takes into account an L1 type penalty, which places a restriction on the combined value of all absolute values for the feature parameters to ensure global optimal performance as well as computing efficiency[[24](#_bookmark35)]. The 2021 IEEE International Conference on Bioinformatics and Biomedicine found that LASSO consistently beats other methods in several key classification parameters, especially AUC, and that the LASSO framework can generate more meaningful feature selection algorithms relative to similar feature selection methods for features[[25](#_bookmark36)]. Furthermore, Neha Shree Maurya et al.[[26](#_bookmark37)] effectively utilized the Lasso algorithm in the field of cancer by extracting signature genes by LASSO and other techniques, leading to the discovery of TMEM236—a new biomarker for the detection of colorectal cancer. Additionally, two separate groups of the first three stages of breast cancer were identified to carry out the differential expression analysis: the first stage vs the subsequent three phases, and early-stage disease versus advanced or metastatic cancer, respectively[[27](#_bookmark38)]. Depending on whether the breast cancer had spread to nearby lymph nodes or somewhere else, it was split into two groups. For the staging groups, differential expression analysis was performed. Following PPI analysis, the final feature genes used for classification were searched for prognostic genes. Breast cancer and breast cancer staging were finally classified using machine learning algorithms such as SVM, random forest, and decision trees. In this case, the results of the model constructed using the features we

extracted produced better results for the early and late diagnosis of breast cancer, and the prognostic genes that were examined provided further recommendations for the treatment of breast cancer.

This work presents a new strategy in breast cancer research by combining systems bi- ology methods and machine learning algorithms—an unusual combination. In addition to providing a greater understanding of the molecular pathways underlying the disease, this dual approach improves the accuracy of cancer diagnosis and staging. Furthermore, the dis- covery of certain proteins (MYH2, MYL1, MYL2, MYH7) and microRNAs (hsa-let-7d-5p) linked to the advancement of breast cancer offers new, prospective biomarkers for diagnosis and therapy, opening the door for innovative therapeutic approaches. The work increases our understanding of breast cancer by applying sophisticated computational algorithms to uncover stage-specific genetic markers using the extensive TCGA dataset, one of the biggest cancer datasets available. Some of the significant contributions of our work are outlined as follows-

1. Diagnosis of breast cancer using gene expression profiling data from the TCGA dataset.
2. Establishment of Protein-Protein Interaction (PPI) network of the differentially Ex- pressed Genes.
3. Classification of different stages of breast cancer using the TCGA dataset with machine learning analysis.

# Materials and Methods

- 1. *Workflow of the Analytical Approach*

To progress the work, we start by acquiring the TCGA data from the GDC portal; RNA- seq data and clinical data from BRCA are collected and downloaded for further progress. To find the marker genes, we performed an analytical approach presented in Figure 1. First we identify the differentially expressed genes (DEGs) in the BRCA dataset using the gene expression counts matrix. After this, some DEGs were amended based on potential DEG selection criteria, which is *|logFC| >* 1*.*0 & *adj.P <* 0*.*05. From the selected DEGs top 10 unregulated and top 10 downregulated DEGs were used for further examination of protein- protein interactions (PPI), ontological and enrichment analysis, regulatory analysis and prediction of drug and chemical compounds. Later on from survival analysis the survival curves of the most influential genes from top 10 up and downregulated genes were found. The identification of signalling and ontological terms was fourth from gene enrichment analysis. Potential hub proteins were identified from the PPI network analysis. TFs(Transcription factors) and miRNAs were identified from the gene regulatory network analysis. Further- more, samples from the TCGA-BRCA project were employed in the experiment for the diagnosis of breast cancer and general health. Following feature extraction, important genes have been selected as diagnostic classification features. Support vector machines (SVM), Random Forest (RF), Decision trees, XGBoost, and AdaBoost are the classifiers used in this experiment by the researchers.


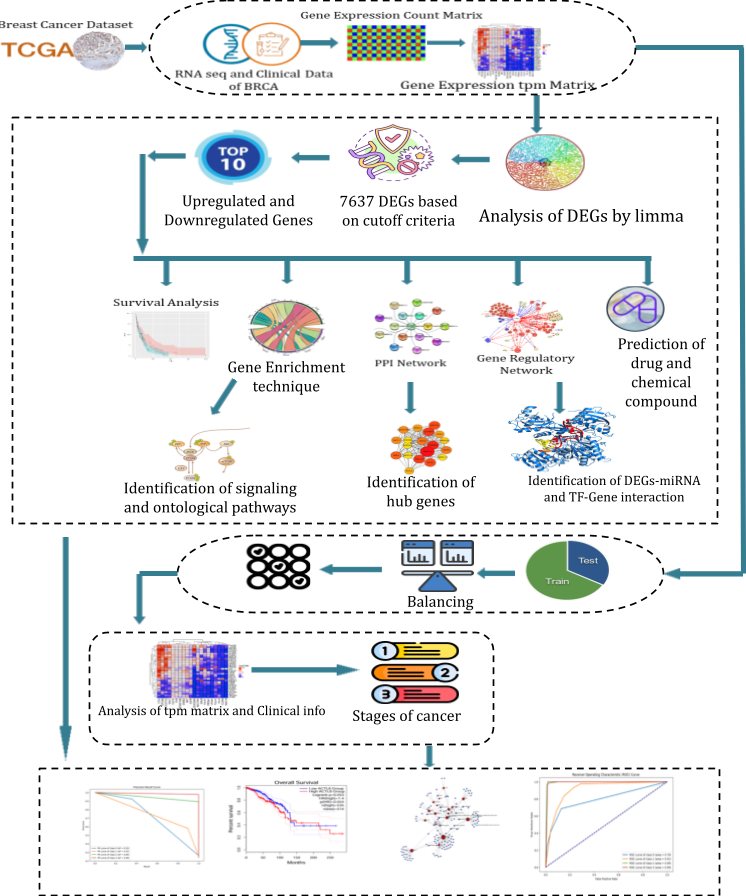


Figure 1: Working flowchart of the analytical study performed in this research.

- 1. *Dataset Description*

We searched the Genomic Data Commons (GDC) Data Portal and collected the Breast cancer (BRCA) gene expression data from TCGA (https://portal.gdc.cancer.gov/repository).

The TCGA official website’s transcriptome gene expression data from the TCGA-BRCA projects were chosen for this study, and from these 1224 samples of 1111 tumor tissue sam- ples and 113 normal tissue samples were obtained for further analysis.

- 1. *Analysis of Differential Expression Gene*

Examining transcriptomic data using differential expression (DE) analysis allows for analyzing variations in gene expression across the entire genome linked to significant biolog- ical conditions [[28](#_bookmark39)]. DEGs play a vital role in order to gain additional biological insights, such as identifying enriched functional pathways, gene ontologies, and PPI(Protein-Protein Interaction) analysis. In this study, the R package limma [[29](#_bookmark40)] with *|logFC| >* 1*.*0 and *adj.P.V al <* 0*.*05 was used to perform the differential expression analysis. Breast cancer tissues were compared to normal tissues to identify genes that were differentially expressed. Later on, the top 10 upregulated and downregulated genes were selected based on the logFC values from the DEGs that were found for analyzing further processes.

- 1. *Analysis of the enrichment of gene set*

A computational and statistical approach known as ”gene set enrichment analysis” is typically used to determine if a collection of determined genes exhibits statistical signifi- cance under various biological circumstances [[30](#_bookmark41)]. The structural and computational data pertaining to gene product-based functions can be found in the GO resources [[31](#_bookmark42)]. Molec- ular function, biological process, and cellular component are the three subcategories of GO that can be used to annotate gene products [[32](#_bookmark43)]. In this research, the online web tool ”Enrichr” was utilized for performing gene enrichment and ontology analysis. Enrichr is a user-friendly, web-based enrichment analysis application that offers a variety of visualization summaries of the combined activities of gene lists [[33](#_bookmark44)]. We assessed the biological relevance of the top ten upregulated and top ten downregulated DEGs of BRCA using signalling and gene ontology terms. In gene enrichment analysis, we selected pathways based on adjusted P-value *<* 0*.*05. The top signalling pathways were found using three databases: KEGG, Bio- Planet, and BioCarta. Top gene ontology terms of molecular function, biological activity, and cellular components were also identified using EnrichR.

- 1. *Identification Hub-bottleneck proteins from PPI Network*

Analysis of protein-protein interactions yields prominent insights into the functions of proteins and is considered the first step in systems biology and drug discovery [[34](#_bookmark45)]. We utilized the top 10 upregulated and downregulated DEGs of BRCA to design a PPI inter- action network using the NetworkAnalyst tool. NetworkAnalyst is a web-based application that allows bench researchers to conduct both simple and complex meta-analyses of gene expression data [[35](#_bookmark46)]. We obtained the hub bottleneck genes from PPI analysis by using Cytohubba in Cytoscape software. Cytoscape is an open-source software framework that can integrate complicated network visualizations with any kind of attribute data [[36](#_bookmark47)]. Cy- tohubba is a Cytoscape plugin that can predict and investigate key nodes and subnetworks inside a given network [[37](#_bookmark48)]. In a complex PPI network, hub nodes are often identified by their extensive connectivity[[38](#_bookmark49)]. Hub nodes play a crucial role in both regulating several

biological processes and maintaining the structural integrity of Protein-Protein Interaction (PPI) networks.

- 1. *Analysis of gene regulatory networks*

To understand the functions of transcription factors (TFs) and microRNAs (miRNAs) which play a significant role in modifying the expression of DEGs linked to breast cancers the gene regulatory network analysis was performed. Comprehensive studies are completed with the help of the online tool NetworkAnalyst[[35](#_bookmark46)], which utilizes databases like the TarBase[[39](#_bookmark50)] and miRTarBase[[40](#_bookmark51)] for DEG-miRNA interactions, and the JASPAR database[[41](#_bookmark52)] for TF- DEG interactions. The target of these experiments is to gain a clear concept of the complex transcriptional and posttranscriptional regulatory mechanisms affecting gene expression in breast cancer. Understanding the molecular mechanisms underlying the pathogenesis of breast cancer is improved by defining these regulatory relationships.

- 1. *Prediction of drugs and chemical compounds*

Using the top 10 upregulated and downregulated genes of BRCA, we were able to create networks of interactions between proteins chemicals, and drugs in this analysis. The com- bined protein-drug and protein-chemical interactions are obtained using the NetworkAnalyst web tool. Analyzing protein-drug interactions is crucial to comprehending the structural fea- tures required for receptor sensitivity [[42](#_bookmark53)]. Protein-chemical interaction analysis is essential for advancing our understanding of biology, accelerating drug discovery efforts, and improv- ing diagnostics and treatments for various diseases.

- 1. *Survival analysis*

One widely used characteristic in research to predict and identify gene signatures in cancer is patient survival analysis, which combines both gene expression and clinical data [[43](#_bookmark54)]. The top 10 upregulated and downregulated genes from DEG analysis were subjected to survival analysis to find genes affecting breast cancer survival. Survival analysis of the top 10 upregulated and top 10 downregulated genes was performed using GEPIA2 ([http://gepia2.cancer-pku.cn/#index).](http://gepia2.cancer-pku.cn/#index)) GEPIA2 is an upgraded web server designed for interactive assessment and large-scale gene analysis. GEPIA2 facilitates the investiga- tion of a particular cancer subtype and subgroup comparison, extending gene expression measurement from the genetic level to the transcripts level [[44](#_bookmark55)].

- 1. *Building the Model*

In this study, 1224 samples from the TCGA-BRCA research were utilized for the di- agnosis and classification of breast cancer stages. These samples included 1111 tumour tissue samples and 113 corresponding control tissue samples. The primary objective was to identify differentially expressed genes (DEGs) to serve as classification features, which were then used to diagnose early and late stages of breast cancer. The dataset comprised 918 samples in the early stage and 306 samples in the late stage. Early-stage breast cancer generally refers to stages I and II and late-stage breast cancer usually encompasses stages III

and IV. Differential analysis was conducted on these samples to identify DEGs, highlight- ing the genes that are significantly upregulated or downregulated in breast cancer tissues compared to control tissues, providing vital information for accurate diagnosis and staging of the disease.

The study employed multiple classifiers to ensure robust and accurate predictions. These classifiers include Gaussian Naive Bayes (GNB), Random Forest (RF), Decision Tree (DT), K-Nearest Neighbors (KNN), XGBoost, and Support Vector Machine (SVM) with RBF Kernel Function. To guarantee that the features were on a similar scale, each classifier started by normalizing the data. This is an important step for enhancing performance, particularly for algorithms that are sensitive to data scale. The dataset was split at random into a test set (30%) and a training set (70%) to give enough information for learning while keeping a sizeable amount for objective assessment. The Synthetic Minority Over-sampling Technique (SMOTE) is used to solve the problem of sample imbalance, particularly between the early and late stages of breast cancer. To balance the class distribution and improve the model’s capacity to generalize and perform well in minority classes, SMOTE creates synthetic examples for the training set. The test set data was used to assess the trained models, and the performance metric was the area under the curve (AUC) of the receiver operating characteristic (ROC) curve. A higher AUC denotes greater performance. The AUC gives an indication of how well the model can discriminate across classes. The outcomes were averaged across 10 runs using cross-validation, a resampling technique used to assess machine learning models on a small data sample, in order to assure robustness and reliability. The decision function for SVM with RBF kernel can be represented as:

With an RBF kernel, the SVM decision function is expressed as:

*f* (*x*) = Σ *α_i_y_i_K*(*x, x_i_*) + *b*

*n*

*i*=1

In this case, the Lagrange multipliers are represented by *α_i_*, the class labels by *y_i_*, the kernel function by *K*, the bias term by *b*, and the support vectors by *x_i_*.

The projected outcome for RF may be shown as:

*y*ˆ = mode(*y*_1_*, y*_2_*, ..., y_n_*)

where the individual tree predictions are denoted by *y*_1_*, y*_2_*, ..., y_n_* and the predicted class is represented by *y*ˆ.

One way to describe the DT decision rule is as:

if *X*_feature_ *≤* threshold then class = left child else class = right child Given the GNB features, the likelihood of a class may be expressed as follows:

*P* (*y|x*_1_*, x*_2_*, ..., x_n_*) =

*P* (*y*) Q*n*

*P* (*x_i_|y*)

*P* (*x*_1_*, x*_2_*, ..., x_n_*)

*i*=1

The XGBoost forecast can be shown as:

300

200

−log10(Adjusted p−value)

100

0

Volcano Plot of DEGs (Upregulated and Downregulated)

|  |  |  |  |  |  |  |  |
| --- | --- | --- | --- | --- | --- | --- | --- |
|  |  |  |  |  |  |  |  |
|  |  |  |  |  |  |  |  |
|  |  |  |  |  |  |  |  |
|  |  |  |  |  |  |  |  |
|  |  |  |  |  |  |  |  |
|  |  |  |  |  |  |  |  |

−5 0 5

Log Fold Change

DEG Status


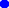
 Downregulated
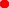
 Upregulated


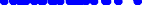


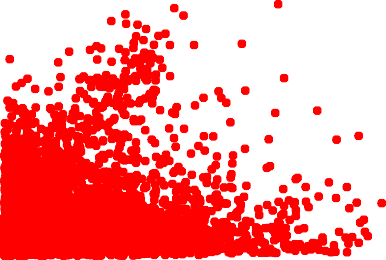

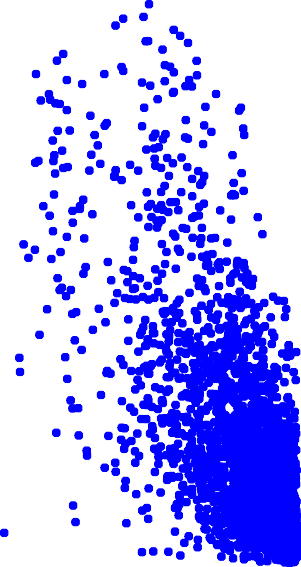


Figure 2: Volcano plot of DEGs. The DEGs are obtained based on criteria of log fold-change (LogFC) less than 1 for downregulated genes greater than 1 for upregulated genes and p-value less than 0.05.

*y*ˆ = Σ *f_m_*(*x*)

*M*

*m*=1

where the forecast of the *m*-th tree is denoted by *f_m_*(*x*) and *M* is the number of trees.

# Results

- 1. *Identification of differentially expressed genes*

7637 genes were ultimately found to be differentially expressed when breast cancer tissues were compared to normal tissues for differential expression analysis. Figure [2](#_bookmark0) depicts the volcano plot of differential expression results where red dots represent upregulated DEGs and blue dots represent downregulated DEGs. Table [1](#_bookmark1) describes the top 10 upregulated and top 10 downregulated differentially expressed genes of the BRCA dataset.

- 1. *Analysis of protein-protein interaction*

In order to obtain hub-bottleneck genes we have generated a protein-protein interaction (PPI) network using the top 10 upregulated and top 10 downregulated genes from the DEGs.

Table 1: Top 10 upregulated and top 10 downregulated differentially expressed genes of BRCA.

| **Gene Symbol** | **Description** | **Regulation** |
| --- | --- | --- |
| UCN3 | Urocortin-3 | Upregulated |
| MUC2 | Mucin 2 |  |
| CGA | Glycoprotein hormones, alpha polypeptide |  |
| CSAG1 | Chondrosarcoma associated gene 1 |  |
| MAGEA12 | MAGE family member A12 |  |
| ACTL8 | Actin like 8 |  |
| MAGEA1 | MAGE family member A1 |  |
| IBSP | Integrin binding sialoprotein |  |
| KLHL1 | kelch like family member 1 |  |
| MAGEA3 | MAGE family member A3 |  |
| MYH2 | Myosin heavy chain 2 | Downregulated |
| CKM | Creatine kinase |  |
| MIR1-1HG | MIR1-1 host gene |  |
| MYL2 | Myosin Light Chain 2 |  |
| MYH7 | Myosin Heavy Chain 7 |  |
| PPP1R3A | Protein Phosphatase 1 Regulatory Subunit 3A |  |
| MYL1 | Myosin Light Chain 1 |  |
| STRIT1 | Small Transmembrane Regulator of Ion Transport 1 |  |
| C10orf71 | Chromosome 10 Open Reading Frame 71 Protein Coding Gene |  |
| NRAP | Nebulin Related Anchoring Protein |  |

Figure [3](#_bookmark2) represents the PPI network of the top 10 upregulated and top 10 downregulated genes of BRCA. We have identified four hub-bottleneck genes, i.e., MYH2, MYL1, MYL2, and MYH7 from the PPI analysis.

- 1. *Identification of signaling and gene ontology terms*

We employed a gene set enrichment analysis to obtain ontological and signalling path- ways. In this analysis, we used the top 10 upregulated and top 10 downregulated genes from the obtained DEGs to identify signaling pathways using five global pathway databases, including KEGG, BioPlanet, and BioCarta. Molecular function, biological process, and cel- lular component were the three classifications from which the ontological pathways were obtained. The top 10 signalling and ontological pathways based on the adj P-value are represented in Tables [2](#_bookmark3) and [3](#_bookmark4) respectively.

- 1. *Identification of DEGs-miRNA and TF-gene interaction*

With the top 10 up and down-regulated DEGs from BRCA, we were able to obtain regulatory components from miRNA-DEGs and TF-DEGs interactions. Figure [4](#_bookmark5) represents the miRNA-DEGs interactions. In Figure [4](#_bookmark5) purple squares represent the miRNAs, and sky blue circles represent the DEGs.

Figure [5](#_bookmark6) represents the TF-DEGs interactions based on the top 10 up and down-regulated genes. In Figure [5](#_bookmark6) indigo blue rhombus shape nodes represent the TFs and the red circular shape nodes represent the associated DEGs. Based on the degree of a node, its dimension is


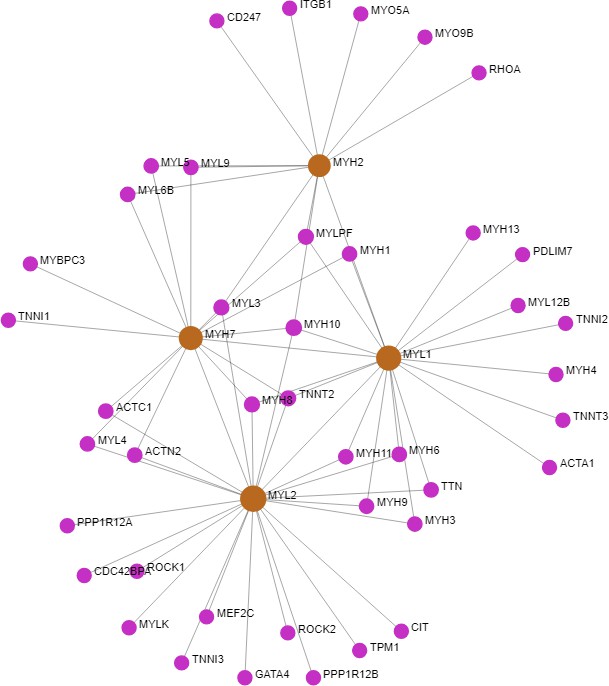


Figure 3: The PPI network of top 10 upregulated and top 10 downregulated genes of BRCA. The bigger circle with different colors represents the top 4 hub proteins.

generated. Four red circular nodes such as MYL1, MYH2, MYL2 and ACTL8 are considered as significant hub genes and four rhombus TFs such as YY1, FOXC1, FOXL1 and MEF2A are considered as regulatory molecules.

- 1. *Identification of protein-drug and protein-chemical interaction*

Figure [6](#_bookmark7) represents the combined protein-drug and protein-chemical network based on the top 10 up and down-regulated genes obtained from BRCA. In Figure [6](#_bookmark7), the red circular nodes indicate drugs and the blue pentangle nodes represent the chemical compounds that have an impact on how genes are expressed. The proteins MAGFA1, MUC2, IBSP, CKM, and MYH2 were considered as the highly expressed therapeutic targets in the combined network.

Table 2: The top 10 signalling pathways of the DEGs obtained from BRCA

| **Category** | **Pathway** | **Genes in the pathway** | **p-value** |
| --- | --- | --- | --- |
| KEGG | Cardiac muscle contraction | MYL2,MYH7 | 0.003377564 |
|  | Hypertrophic cardiomyopathy | MYL2, MYH7 | 0.003609434 |
|  | Dilated cardiomyopathy | MYL2,MYH7 | 0.004094897 |
|  | Adrenergic signaling in cardiomyocytes | MYL2,MYH7 | 0.009716646 |
|  | Focal adhesion | IBSP,MYL2 | 0.016953582 |
|  | Neuroactive ligand-receptor interaction | UCN3,CGA | 0.044995524 |
|  | Arginine and proline metabolism | CKM | 0.04885255 |
|  | Ovarian steroidogenesis | CGA | 0.049806079 |
|  | Autoimmune thyroid disease | CGA | 0.051710413 |
|  | Regulation of lipolysis in adipocytes | CGA | 0.053611122 |
| BioPlanet | Tight junction | MYH2,MYL2,MYH7 | 3.02E-04 |
|  | Striated muscle contraction | MYL1,MYL2 | 6.54E-04 |
|  | Muscle contraction | MYL1,MYL2 | 0.001086179 |
|  | Retinoblastoma protein regulation | CKM,MYL1 | 0.001961104 |
|  | NFAT involvement in hypertrophy of the heart | MYH2,MYL2 | 0.002141024 |
|  | Viral myocarditis | MYH2,MYH7 | 0.002265166 |
|  | Cardiac muscle contraction | MYL2,MYH7 | 0.00286498 |
|  | Dilated cardiomyopathy | MYL2,MYH7 | 0.0044345 |
|  | Glycoprotein hormones | CGA | 0.004990441 |
|  | SARS coronavirus protease | CKM | 0.006979996 |
| BioCarta | Regulators of Bone Mineralization Homo sapiens h npp1Pathway | IBSP | 0.01094778 |
|  | PKC-catalyzed phosphorylation of inhibitory phosphoprotein of myosin phosphatase Homo sapiens h myosinPathway | MYL2 | 0.020801468 |
|  | CCR3 signaling in Eosinophils Homo sapiens h CCR3Pathway | MYL2 | 0.022760983 |
|  | ALK in cardiac myocytes Homo sapiens h alkPathway | MYL2 | 0.026668846 |
|  | NFkB activation by Nontypeable Hemophilus influenzae Homo sapiens h nthiPathway | MYL2 | 0.028617205 |
|  | Rho cell motility signaling pathway Homo sapiens h rhoPathway | MYL2 | 0.0315328 |
|  | Rac 1 cell motility signaling pathway Homo sapiens h rac1Pathway | MYL2 | 0.03540733 |
|  | Trefoil Factors Initiate Mucosal Healing Homo sapiens h tffPathway | MUC2 | 0.03540733 |
|  | NFAT and Hypertrophy of the heart Homo sapiens h nfatPathway | MYL2 | 0.043112259 |

- 1. *Survival Analysis Results*

Survival analysis revealed that only 4 genes among the top 10 upregulated genes and downregulated genes found from DEG analysis were associated with the prognosis of breast cancer, namely ACTL8, CGA, IBSP and MUC2 genes and their survival curves are shown in Figure [7](#_bookmark8).

- 1. *Results of Different Machine Learning Models*

Table [4](#_bookmark11) shows the assessment metrics for several machine learning models used to classify different stages of breast cancer. Each model was evaluated using numerous performance indicators, including accuracy, precision, recall, F1 score, and specificity. The stages of breast cancer were divided into three categories: I-II, III, IV, V; I, II-III, IV, V; and I, II, III-IV, V, allowing for a thorough examination across stages. Notably, Random Forest (RF) and XGBoost consistently achieved good accuracy, precision, recall, and F1 scores across different stages of breast cancer. These models’ accuracy evaluations, which indicate their capacity to distinguish between different cancer stages, varied from 85.51% to 97.19% for RF and from 85.51% to 95.23% for XGBoost. Excellent accuracy ratings were generated by both RF and XGBoost, ranging from 85.58% to 97.20% for RF and 85.59% to 95.34% for XGBoost. These results show that both methods can consistently recognize real positive circumstances. The high recall figures (85.51% to 97.19% for RF and 85.51% to 95.23% for XGBoost) show that both techniques were successful in gathering all positive examples. Moreover, RF and XGBoost routinely had high F1 ratings, which show the harmonic mean of accuracy and recall, indicating their overall efficacy.

With an accuracy range of 63.30% to 85.19%, the Support Vector Machine (SVM) also

performed well, most notably in differentiating between phases I–II and III, IV, and V.

Table 3: The top 10 gene ontology terms of the DEGs obtained from BRCA

| **Category** | **Pathway** | **Genes in the pathway** | **p-value** |
| --- | --- | --- | --- |
| Molecular function | Histone Deacetylase Binding | MAGEA12,MAGEA1,MAGEA3 | 1.26E-04 |
|  | Actin Binding | MYL2,NRAP,KLHL1 | 7.66E-04 |
|  | Myosin Heavy Chain Binding | MYL2 | 0.005985691 |
|  | Cuprous Ion Binding | MUC2 | 0.007973357 |
|  | Caspase Binding | MAGEA3 | 0.012926021 |
|  | Peptide Hormone Receptor Binding | UCN3 | 0.012926021 |
|  | Muscle Alpha-Actinin Binding | NRAP | 0.013913732 |
|  | Neuropeptide Receptor Binding | UCN3 | 0.016871235 |
|  | Protein Phosphatase 1 Binding | PPP1R3A | 0.016871235 |
|  | Actin Monomer Binding | MYL2 | 0.022760983 |
|  | Alpha-Actinin Binding | NRAP | 0.022760983 |
|  | Copper Ion Binding | MUC2 | 0.044071254 |
|  | Myosin Binding | MYL2 | 0.05266122 |
|  | Hormone Activity | CGA | 0.071488248 |
| Biological process | Actin-Myosin Filament Sliding | MYH2,MYL1,MYH7 | 1.87E-07 |
|  | Muscle Filament Sliding | MYL1,MYH7 | 4.25E-05 |
|  | Cardiac Myofibril Assembly | MYL2,NRAP | 9.90E-05 |
|  | Muscle Contraction | MYH2,MYL1,MYH7 | 1.08E-04 |
|  | Ventricular Cardiac Muscle Tissue Development | MYL2,MYH7 | 2.81E-04 |
|  | Cardiac Muscle Tissue Morphogenesis | MYL2,MYH7 | 4.34E-04 |
|  | Cardiac Muscle Contraction | MYL2,MYH7 | 4.92E-04 |
|  | Ventricular Cardiac Muscle Tissue Morphogenesis | MYL2,MYH7 | 4.92E-04 |
|  | Positive Regulation Of Intracellular Transport | MYL1,STRIT1 | 6.89E-04 |
|  | Heart Contraction | MYL2,MYH7 | 7.61E-04 |
|  | Cardiac Ventricle Morphogenesis | MYL2,MYH7 | 7.99E-04 |
|  | Myofibril Assembly | MYL2,MYH7 | 9.58E-04 |
|  | Striated Muscle Contraction | MYL2,MYH7 | 0.001467055 |
|  | Host-Mediated Regulation Of Intestinal Microbiota Composition | MUC2 | 0.005985691 |
| Cellular component | Myofibril | MYH2,MYL1,MYL2,MYH7 | 1.70E-08 |
|  | Muscle Myosin Complex | MYH2,MYL1,MYH7 | 3.09E-07 |
|  | Myosin Filament | MYH2,MYH7 | 9.90E-05 |
|  | Supramolecular Fiber | MYH2,MYH7 | 5.86E-04 |
|  | Golgi Lumen | MUC2,CGA | 0.0044345 |
|  | Sarcoplasmic Reticulum Membrane | STRIT1 | 0.026668846 |
|  | Intercalated Disc | NRAP | 0.03056186 |
|  | Cell-Cell Junction | MYH2,NRAP | 0.035451148 |
|  | Actin Cytoskeleton | MYL2,ACTL8 | 0.04171588 |
|  | Sarcoplasmic Reticulum | STRIT1 | 0.044071254 |
|  | Caveola | MYL1 | 0.06023514 |
|  | Actin Filament | ACTL8 | 0.068687033 |


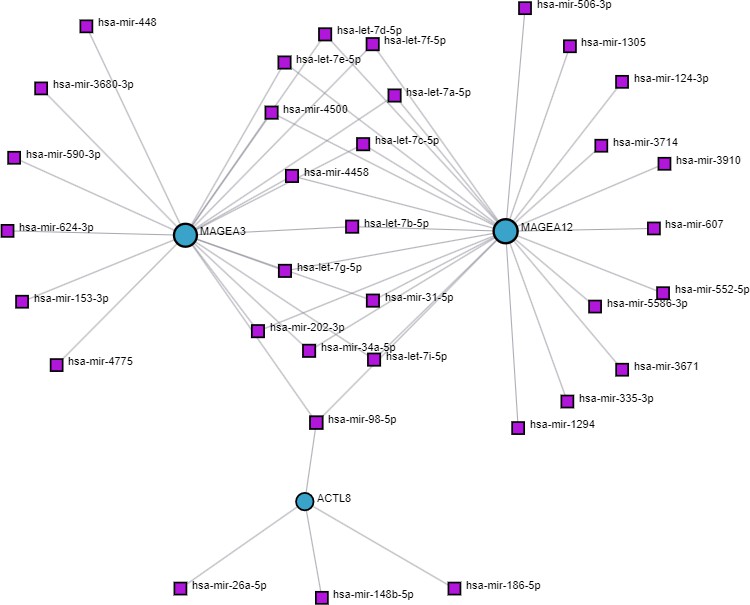


Figure 4: miRNA-gene interaction regulatory network. Target regulatory molecules are represented by purple square nodes, while associated genes are represented by sky-blue circular nodes.

But compared to RF and XGBoost, SVM has a lower specificity, suggesting that it would have trouble correctly identifying actual negative scenarios. Phase-by-phase variations in the decision tree’s (DT) accuracy ranged from 67.22% to 85.19%. While DT had poorer accuracy than RF and XGBoost, it nevertheless obtained acceptable performance metrics, demonstrating its potential value in specific applications. Gaussian Naive Bayes (GNB) showed lower accuracy than other models, ranging from 63.30% to 83.74%. This shows that GNB may be less efficient at reflecting the complexity of breast cancer staging than more advanced models such as RF and XGBoost.K-Nearest Neighbors (KNN) achieved reasonable accuracy, ranging from 77.77% to 80.63%. While KNN demonstrated lesser accuracy than RF and XGBoost, its performance was nonetheless respectable, demonstrating its potential use in certain scenarios.

Overall, the results show that RF and XGBoost are successful in properly classifying different stages of breast cancer, implying that they might be used in clinical practice for precise diagnosis and treatment planning. Figure [8](#_bookmark9) displays the precision-recall curve, while Figure [9](#_bookmark10) displays the ROC curve for the four distinct models.


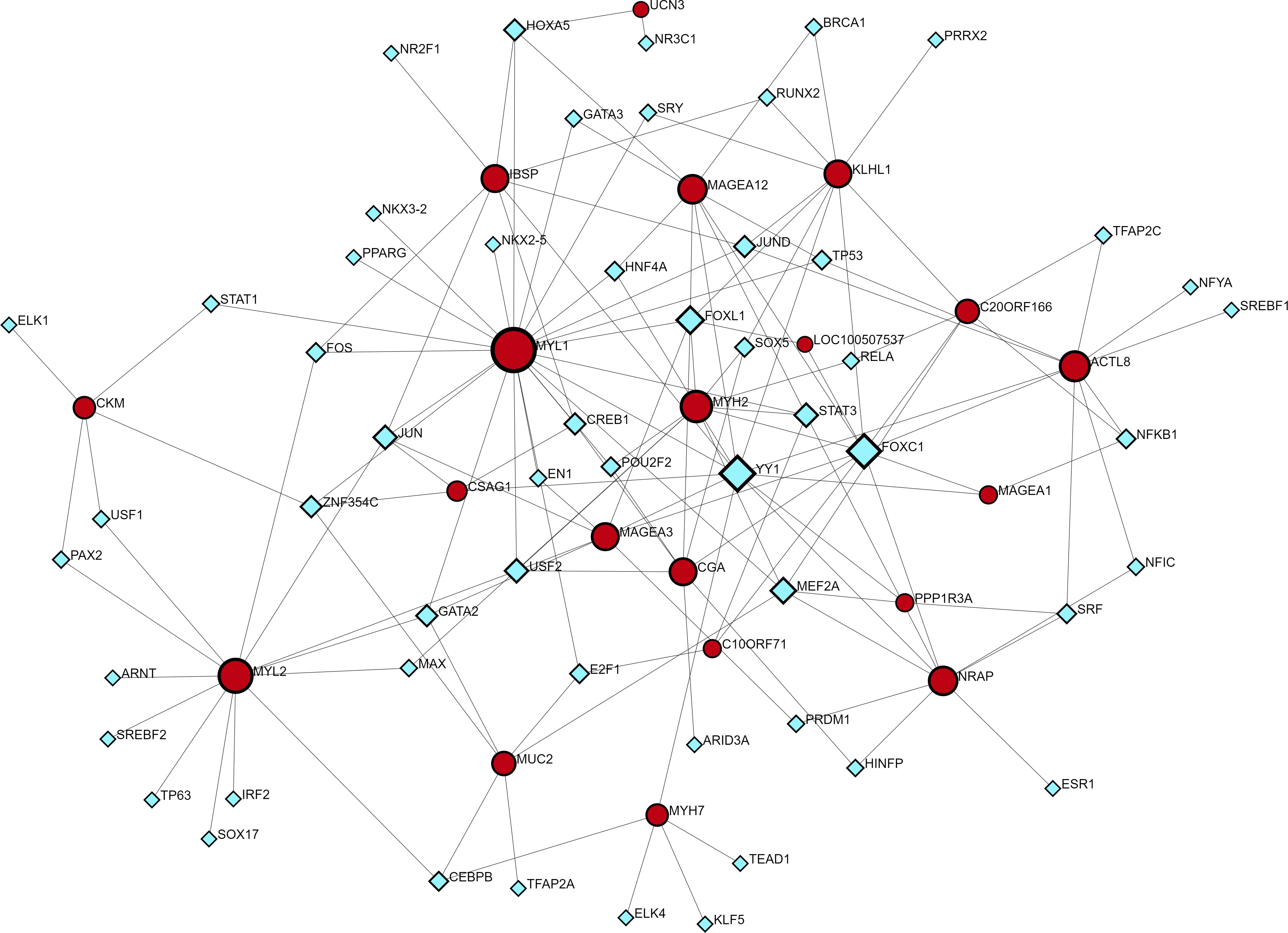


Figure 5: TF-gene interaction regulatory network. Indigo blue square nodes indicate target regulatory molecules(TFs) and red circular shape nodes represent the associated DEGs

# Discussion

In women globally, breast cancer is the primary cause of cancer-related death. In both developed and developing nations, it comes in second place among the main causes of cancer-related deaths. Treatment effectiveness dramatically reduces metastasis and post- carcinogenesis, highlighting how crucial early identification is. Not only does prompt diag- nosis increase patient survival rates, but it also makes it easier to put therapies into place that can reduce morbidity and increase overall survival rates [[45](#_bookmark56)]. Breast cancer screening in various European countries is mostly performed via mammography or breast magnetic resonance imaging (MRI), although these technologies present problems. Although breast magnetic resonance imaging (MRI) can be costly and has certain drawbacks, radiologists’ experience is crucial in interpreting mammograms. Both screening techniques are widely used, however it is important to carefully weigh their advantages and disadvantages [[46](#_bookmark57)]. In this study, we focus on identifying and classifying breast cancer using system biology and machine learning approaches. We have studied the breast cancer gene expression data from TCGA datasets. First of all, we utilized the differentially expressed genes (DEGs) of the gene expression data of TCGA datasets and then, we found 20 DEGs including 10 upregulated and 10 downregulated genes of breast cancer (Table 1).To discover prospective therapeutic targets, we examined differentially expressed breast cancer genes using protein-protein in-


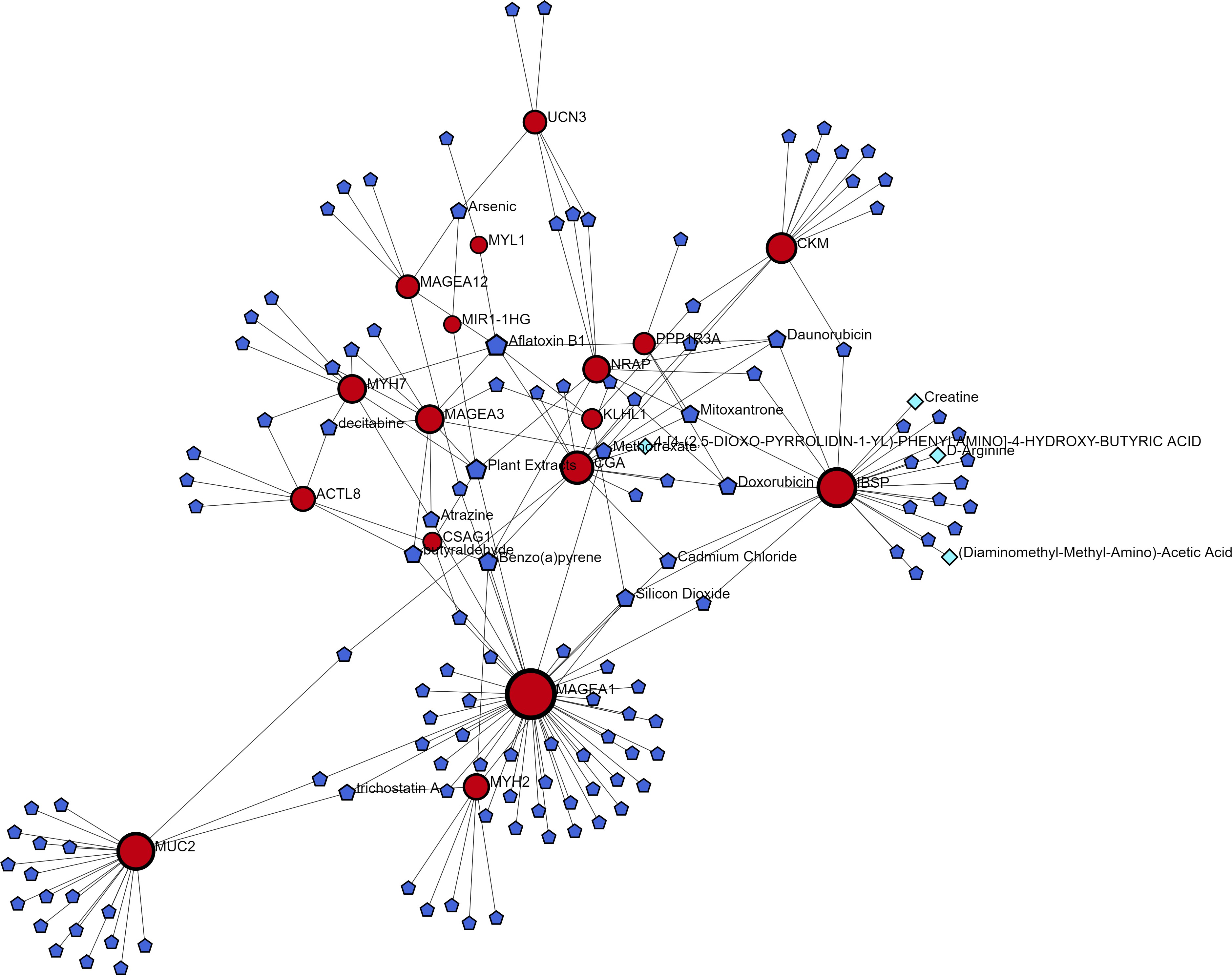


Figure 6: Combined protein-drug and protein-chemical interaction network. Blue color pentangle nodes indicate chemical compounds and pink color rhombus nodes indicate drug regulatory molecules.

teraction, molecular signalling pathway, ontology, and regulatory analysis. Similarly, system biology studies were done to study the molecular markers and therapeutic targets by Barua, Akhtar and Omit [[47](#_bookmark58), [48](#_bookmark59), [49](#_bookmark60)].

We utilized gene enrichment analysis to obtain the responsible genes of breast cancer by discovering gene ontologies and pathways. We identified 20 DEGs using gene ontological ex- ploration based on P-value to obtain insight into the molecular importance of breast cancer. The three types of GO analysis such as molecular function (molecular level performance), biological process (biological activities), and cellular component (gene regulatory activities) were employed from the GO database using Enrichr as an annotation source [[50](#_bookmark61)]. In the molecular function, Histone Deacetylase Binding and Actin Binding activity are significant among the top GO terms. Actin-myosin filament Sliding and Muscle Filament Sliding for biological processes and Myofibril and Muscle Myosin Complex for cellular components are among the top GO terms.

Myofibrils are the complex structures found inside muscle cells. They are essential for

enabling muscular contraction, which is a basic mechanism that is necessary for movement and body function. This process also greatly contributes to general physiological balance. Several muscle diseases and ailments that impair movement and physical well-being may

Overall Survival Overall Survival


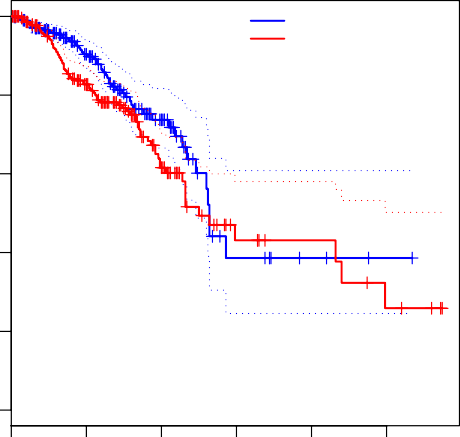

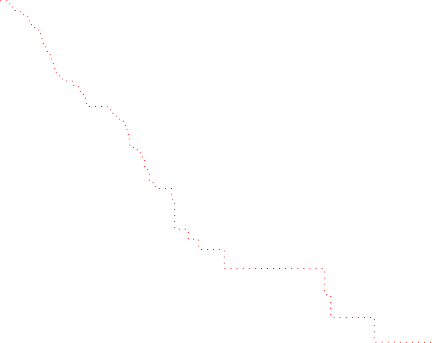


Low ACTL8 Group High ACTL8 Group Logrank p=0.053

HR(high)=1.4 p(HR)=0.054

n(high)=535 n(low)=514


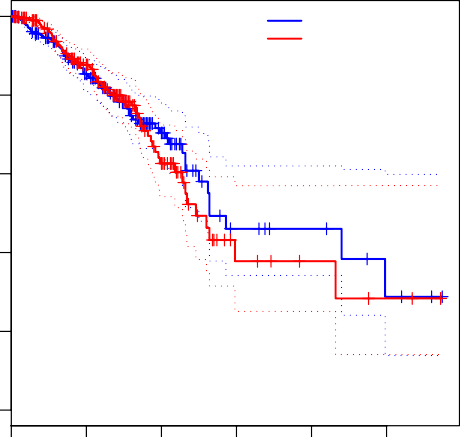


Low CGA Group High CGA Group Logrank p=0.96

HR(high)=0.99 p(HR)=0.96

n(high)=530 n(low)=535

Percent survival

0.6

0.8

1.0

Percent survival

0.6

0.8

1.0

0 50 100 150 200 250

0.0

0.2

0.4

0.0

0.2

0.4

Months

0 50 100 150 200 250

Months

Overall Survival Overall Survival


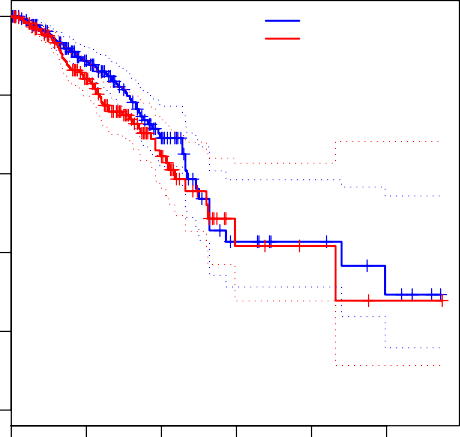


Low IBSP Group High IBSP Group Logrank p=0.16

HR(high)=1.3 p(HR)=0.16

n(high)=533 n(low)=535


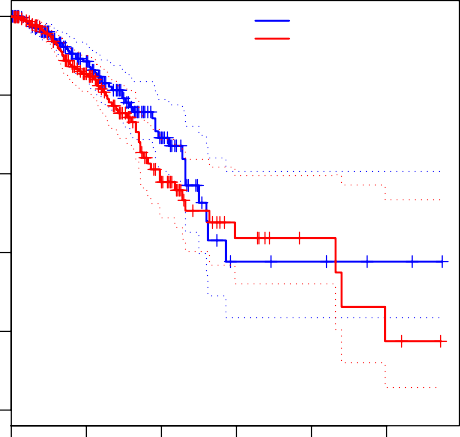


Low MUC2 Group High MUC2 Group Logrank p=0.26

HR(high)=1.2 p(HR)=0.26

n(high)=475 n(low)=387

Percent survival

0.4

0.6

0.8

1.0

Percent survival

0.4

0.6

0.8

1.0

| 0 | 50 | 100 150 200 | 250 | 0 | 50 | 100 150 200 250 |
| --- | --- | --- | --- | --- | --- | --- |
|  |  | Months |  |  |  | Months |

Figure 7: Overall Survival rate of the genes ACTL8, CGA, IBSP and MUC2

0.0

0.2

0.0

0.2

be related to myofibril dysfunction or dysregulation[[51](#_bookmark62)]. Histone deacetylases (HDACs) in breast cancer regulate gene expression by altering histone proteins. This can affect the expression of genes involved in cell growth, metastasis, and medication resistance[[52](#_bookmark63)]. HDAC inhibitors have shown promise as possible therapies because they reverse these processes and make cancer cells more susceptible to therapy. Another study showed that alterations in cytoskeletal dynamics, including actin-myosin interactions, can indirectly influence various aspects of breast cancer progression [[53](#_bookmark64), [54](#_bookmark65)].

Enrichment analysis is an important tool for identifying correlations unique to breast cancer and other disorders [[55](#_bookmark66), [56](#_bookmark67)]. The KEGG pathway of the DEGs revealed the top four pathways Cardiac muscle contraction, Hypertrophic cardiomyopathy, Dilated cardiomyopa-


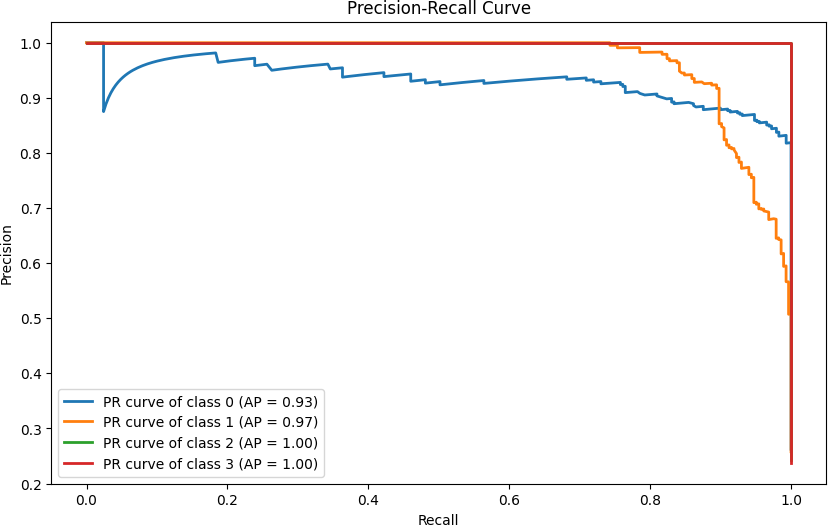

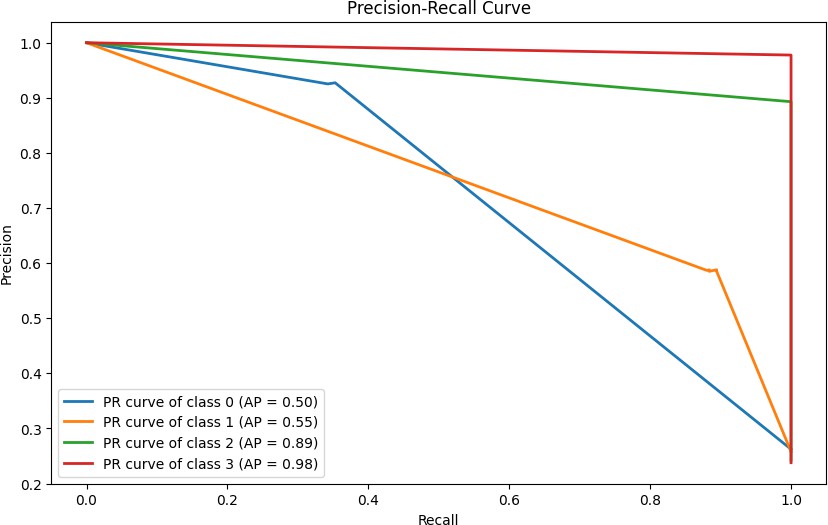


(a) Precision-Recall Curve(RF) (b) Precision-Recall Curve(GNB)


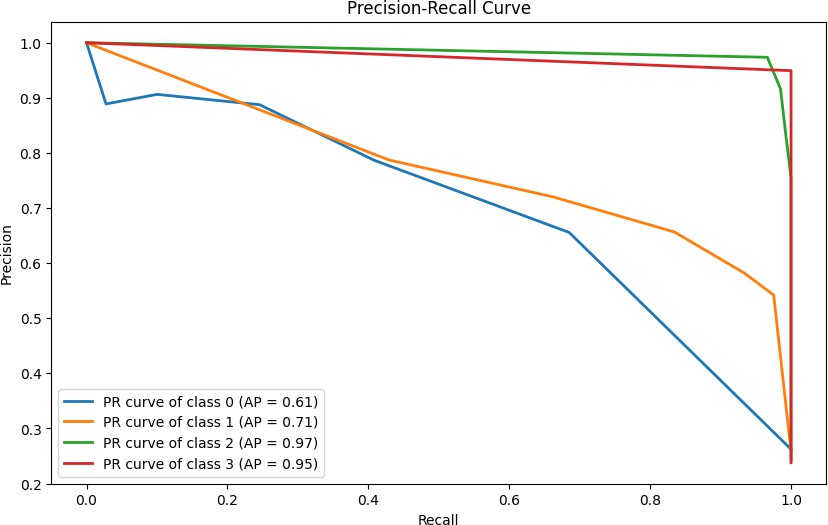

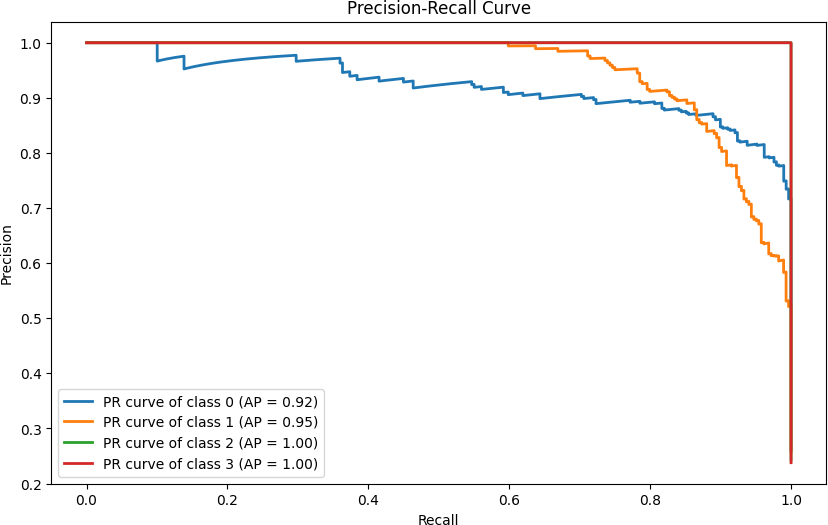


(c) Precision-Recall Curve(KNN) (d) Precision-Recall Curve(XGB)

Figure 8: Precision-Recall Curve of machine learning models RF(Random Forest), GNB(Gaussian Naive Bayes) , KNN (K-Nearest Neighbors) and XGB(XGBoost).

thy, and Adrenergic signaling in cardiomyocytes. In a study Cardiac muscle contraction pathways could potentially impact breast cancer growth by common molecular processes or systemic consequences, as shown by the linked signaling networks in cancer-associated fi- broblasts (CAFs) and the tumor microenvironment (TME)[[57](#_bookmark68)]. According to some research, there seems to be a link between the advancement of breast cancer and chronic stress, which can trigger adrenergic signaling pathways [[58](#_bookmark69)]. In addition, Tight junction and Striated mus- cle contraction for BioPlanet pathway, and Regulators of Bone Mineralization Homo sapiens h npp1Pathway and PKC-catalyzed phosphorylation of inhibitory phosphoprotein of myosin phosphatase Homo sapiens h myosinPathway for BioCarta pathway were revealed as the top significant pathways (Table 2). It has been found that frequent exercise, which burns calo- ries through muscular contraction and may affect the metabolism of creatine, lowers the risk of breast cancer[[59](#_bookmark70), [60](#_bookmark71)]. Moreover, abnormal metabolism including the metabolism of crea- tine may contribute to the advancement of cancer[[61](#_bookmark72), [62](#_bookmark73)]. The Striated Muscle Contraction Pathway, PtdIns 4 5 P2 In Cytokinesis Pathway, and Osteoblast Signaling are all thought to have a role in breast cancer. While these pathways are largely concerned with muscle function, cell division, and bone growth, there may be indirect links or common regulatory


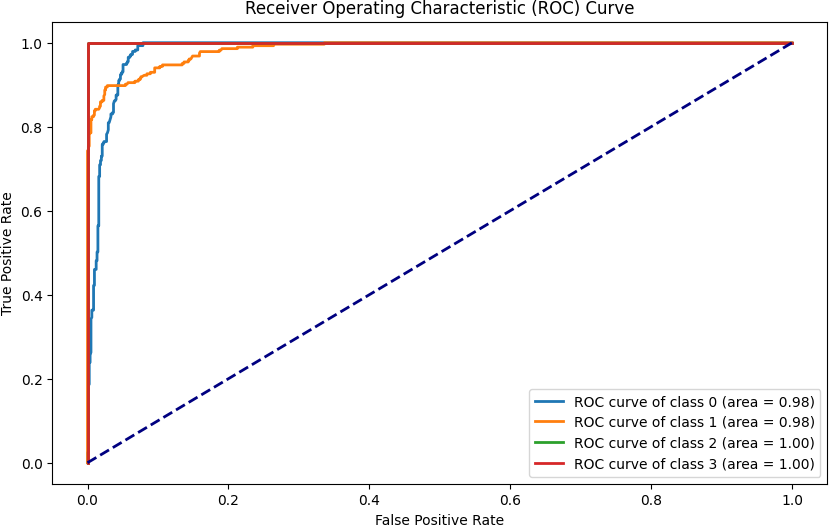

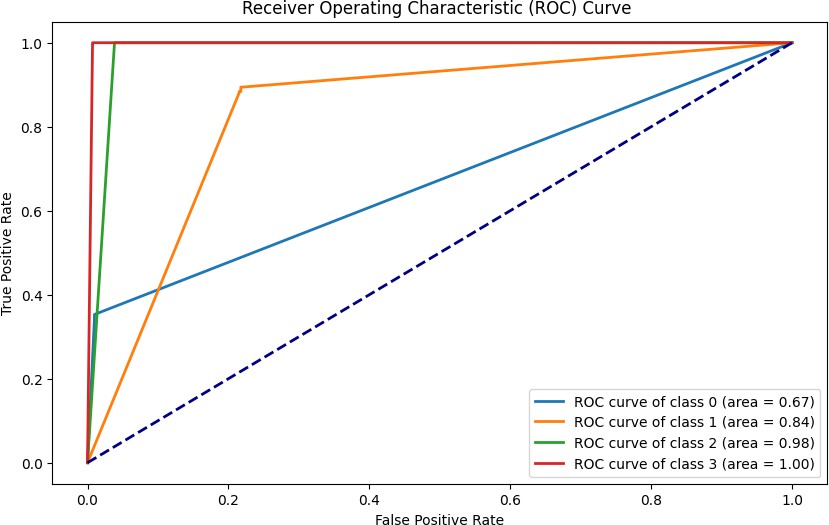


(a) ROC Curve(RF) (b) ROC Curve(GNB)


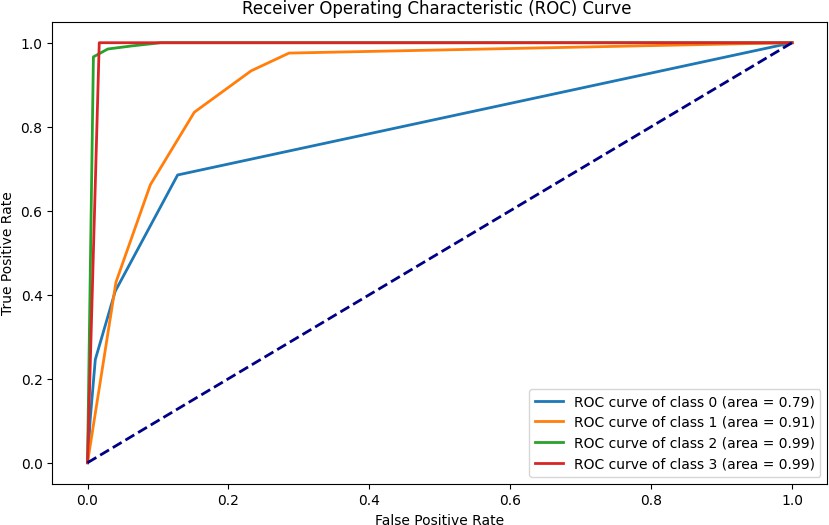

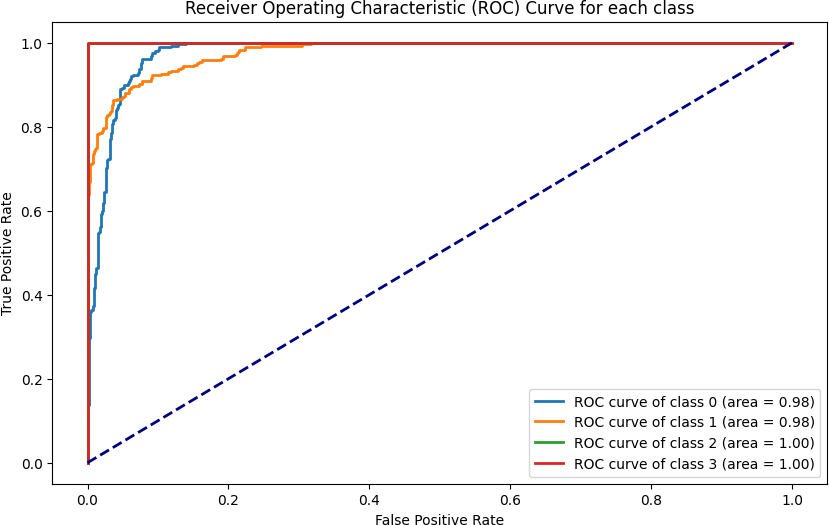


(c) ROC Curve(KNN) (d) ROC Curve(XGB)

Figure 9: Receiver Operating Characteristic(ROC) Curve of machine learning models RF, GNB, KNN and XGB.

mechanisms with breast cancer. Dysregulation of cell division signaling pathways, such as cytokinesis, has been linked to the development of cancer. Furthermore, modifications in bone signaling pathways may impact the bone microenvironment, influencing the course of breast cancer bone metastases[[63](#_bookmark74), [64](#_bookmark75)].

Analysis of protein-protein networks is a crucial method for determining the processes behind the development of illness [[65](#_bookmark76), [66](#_bookmark77)]. In order to acquire hub proteins, we built a network of interactions between proteins. The PPI analysis revealed four hub proteins that are MYH2, MYL1, MYL2, and MYH7. It’s worth noting that changes in numerous cytoskeleton components, such as myosin and myosin-associated proteins, have been linked to cancer development, particularly breast cancer. These modifications can impact cell motility, invasion, and metastasis, which are important factors in cancer growth[[67](#_bookmark78), [68](#_bookmark79)].

MicroRNAs (miRNAs) and transcription factors (TFs) regulate gene expression through post-transcriptional and transcriptional mechanisms. The dysregulation of microRNAs (miRNAs) and transcription factors (TFs) has emerged as a critical mechanism in breast cancer pathogenesis, impacting multiple aspects of tumour initiation, development, and metastasis. Several studies have highlighted the deregulation of certain microRNAs in

Table 4: Model Evaluation Metrics

| Stage | Model | Accuracy | Precision | Recall | F1 | Specificity |
| --- | --- | --- | --- | --- | --- | --- |
|  | RF | **94.46**% | 94*.*61% | 94*.*46% | 94*.*45% | 93*.*77% |
|  | SVM | 85*.*19% | 84*.*19% | 85*.*19% | 84*.*98% | 77*.*16% |
| I-II,III,IV,V | DT | 83*.*66% | 83*.*44% | 84*.*66% | 83*.*47% | 67*.*50% |
|  | GaussianNB | 79*.*76% | 68*.*02% | 73*.*33% | 69*.*42% | 36*.*23% |
|  | KNN | 80*.*63% | 68*.*02% | 73*.*33% | 69*.*42% | 60*.*89% |
|  | XGBoost | 93*.*01% | 93*.*27% | 93*.*01% | 92*.*99% | 92*.*38% |
|  | RF | **97.19**% | 97*.*20% | 97*.*19% | 97*.*18% | 92*.*88% |
|  | SVM | 77*.*95% | 77*.*95% | 77*.*95% | 76*.*56% | 83*.*90% |
| I,II-III,IV,V | DT | 85*.*19% | 84*.*19% | 85*.*19% | 84*.*98% | 77*.*16% |
|  | GaussianNB | 83*.*74% | 86*.*25% | 83*.*74% | 82*.*65% | 97*.*36% |
|  | KNN | 77*.*77% | 80*.*23% | 77*.*70% | 73*.*46% | 96*.*08% |
|  | XGBoost | 95*.*23% | 95*.*34% | 95*.*23% | 95*.*23% | 87*.*45% |
|  | RF | **85.51**% | 85*.*58% | 85*.*51% | 85*.*53% | 89*.*71% |
|  | SVM | 63*.*30% | 63*.*43% | 63*.*30% | 61*.*87% | 82*.*69% |
| I,II,III-IV,V | DT | 67*.*22% | 67*.*00% | 67*.*22% | 67*.*00% | 74*.*44% |
|  | GaussianNB | 74*.*82% | 77*.*85% | 74*.*82% | 72*.*67% | 96*.*13% |
|  | XGBoost | **85.51**% | 85*.*84% | 85*.*51% | 85*.*59% | 88*.*78% |

breast cancer, such as miR-21, miR-155, and miR-221, which are typically overexpressed and linked with poor prognosis[[69](#_bookmark80), [70](#_bookmark81)]. By specifically targeting oncogenes or important tumour suppressor genes, these miRNAs can alter vital signaling pathways that are in- volved in invasion, apoptosis, and cell proliferation. Furthermore, abnormal expression of TFs, including members of the E2F, FOX, and AP-1 families, has been linked to breast cancer development[[71](#_bookmark82), [72](#_bookmark83)]. TFs control the expression of genes involved in a variety of biological functions. When TFs are dysregulated, normal gene expression patterns can be disrupted, which can contribute to the development of cancer.We identified the top signif- icant miRNA targets (hsa-let-7d-5p, hsa-mir-4500, hsa-mir-34a-5p, hsa-let-7a-5p and hsa- let-7c-5p) which may be interconnected with pathways of breast cancer (Figure 3). The target miRNAs may be regarded biomarkers and therapeutic targets to treat breast cancer [[73](#_bookmark84), [74](#_bookmark85)]. The top significant regulatory TFs ( YY1, FOXC1, FOXL1 and MEF2A) may be re- sponsible for the related pathways of breast cancer cellular process of disease development. Among the discovered TFs, YY1 has been implicated in tumor aggressiveness and medi- cation resistance through regulation of cell cycle control and metastasis-related genes[[75](#_bookmark86)]. FOXC1, on the other hand, promotes tumor growth and metastasis by regulating genes associated with epithelial-mesenchymal transition (EMT) and angiogenesis, contributing to poor clinical outcomes[[76](#_bookmark87)]. In another study[[77](#_bookmark88)], researchers discovered that while the spe- cific role of FOXL1 in breast cancer remains unclear, accumulating evidence shows that it may have tumor-suppressive activities, reducing proliferation and invasion in breast cancer

cells. Meanwhile, MEF2A has been linked in boosting tumor development and metastasis via modulating genes involved in cell proliferation and survival, indicating a bad prognosis in breast cancer patients[[78](#_bookmark89)].

Apart from clarifying the functions of transcription factors in breast cancer, protein- chemical interaction research has discovered other possible targets for treatment. Because it inhibits dihydrofolate reductase and messes with DNA synthesis, methotrexate, a com- monly used chemotherapeutic treatment, has demonstrated success in treating a variety of malignancies, including breast cancer [[79](#_bookmark90)]. Because of its capacity to cause DNA damage and encourage carcinogenesis, benzopyrene, a polycyclic aromatic hydrocarbon present in tobacco smoke, has been linked to the development of breast cancer [[80](#_bookmark91)]. 4-[4-(2,5-DIOXO- PYRROLDIN-1-YL)-PHENYLAMINO] is the compound. Despite not having been well researched, -4-HYDROXY-BUTYRIC ACID shows promise as a therapeutic agent since it targets particular biochemical pathways that are implicated in the advancement of breast cancer [[81](#_bookmark92)].

Furthermore, machine learning analysis is implemented to improve the accuracy of breast

cancer stage classifications. Notably, Random Forest (RF) and XGBoost consistently de- livered excellent accuracy, precision, recall, and F1 scores throughout all stages, with RF ranging from 85.51% to 97.20% and XGBoost from 85.51% to 95.34%. SVM was effective in distinguishing between phases I-II and III, IV, and V, with accuracy ranging from 63.30% to 85.19%. However, SVM had poorer specificity than RF and XGBoost. Decision Tree (DT) performance varied, although Gaussian Naive Bayes (GNB) accuracy was lower. K-Nearest Neighbors (KNN) demonstrated reasonable accuracy. Overall, RF and XGBoost indicate potential for therapeutic usage in precise breast cancer staging.

This research has several benefits for the identification, categorization, and staging of breast cancer. The TCGA dataset combines bioinformatics and machine learning to offer a thorough examination of molecular markers and cancer development. High diagnosis accu- racy (97.19% and 95.23%, respectively) is obtained by the application of machine learning models like Random Forest and XGBoost, which may be very helpful for clinical practice. More focused treatment approaches are also made possible by the discovery of possible ther- apeutic targets by the examination of signaling cascades, protein-protein interactions, and differentially expressed genes (DEGs). Finding important proteins and microRNAs con- nected to the development of cancer also provides useful biomarkers for early identification and individualized treatment. Through the implementation of systems biology methods, the research expands our knowledge of the molecular pathways underlying breast cancer and helps to advance personalized medicine strategies that customize treatment regimens based on the unique characteristics of each patient. All things considered, this research offers a strong foundation for improving breast cancer detection, staging, and treatment advancement.

# Conclusions

The complete investigation of breast cancer utilizing systems biology and machine learn- ing methodologies has revealed important information about disease processes and prospec-

tive treatment targets. Significant molecular activities such as histone deacetylase binding and actin binding were found among the elevated genes, suggesting that they have active roles in cancer development. Similarly, downregulated genes were linked to key biological processes such as muscle filament sliding, indicating a possible imbalance in cellular func- tions. Enrichment analysis helped to understand the pathways that stimulate breast cancer development. The main pathways identified, including heart muscle contraction and adren- ergic signaling, indicate possible linkages between cancer and systemic processes such as chronic stress. Furthermore, the enrichment of pathways associated with muscle contraction and cytokinesis emphasizes the role of cytoskeletal dynamics in cancer development, open- ing up new possibilities for therapeutic intervention. The protein-protein interaction study revealed hub proteins such as MYH2 and MYH7, demonstrating the role of cytoskeletal components in breast cancer aetiology. MicroRNA and transcription factor studies revealed dysregulation of key regulators such as hsa-let-7d-5p and YY1, indicating their potential as diagnostic indicators and therapeutic targets. Furthermore, the discovery of chemicals with therapeutic potential, such as methotrexate and 4-HYDROXY-BUTYRIC ACID, empha- sizes the need to address particular biochemical pathways in cancer treatment. In addition, machine learning studies showed that models such as Random Forest (RF) and XGBoost can reliably detect breast cancer stages, with RF attaining an accuracy range of 85.51% to 97.20% and XGBoost ranging from 85.51% to 95.34%. The Support Vector Machine (SVM) has shown success in differentiating between phases I-II and III, IV, and V, with an accuracy range of 63.30% to 85.19%. However, SVM has lower specificity than RF and XGBoost.

One key limitation of the study is the lack of clinical trials to validate the findings.

While the machine learning models show high accuracy in cancer classification, their clinical applicability requires further testing to confirm their effectiveness in real-world settings. Additionally, the study primarily focuses on specific subtypes and stages of breast cancer, necessitating further research to assess the methodology’s effectiveness across a broader range of breast cancer types and diverse patient populations. Another key limitation of this study is the lack of clinical trials to validate the findings, as the machine learning models, while demonstrating high accuracy in classification, require further testing to assess their clinical applicability. Additionally, the study’s focus on specific subtypes and stages of breast cancer limits its scope, and further research is needed to evaluate the methodology’s effectiveness across a wider range of breast cancer types and diverse patient populations. Despite the use of SMOTE to address class imbalance, the dataset’s imbalance, particularly in later-stage cancers, may still impact model performance and its ability to generalize effectively. The study’s research findings can be used in laboratory studies to better understand potential therapeutic targets for breast cancer treatments.

# Acknowledgements Availability of data

The datasets for this study were collected from the Genomic Data Commons (GDC) Data

Portal and the Breast cancer (BRCA) gene expression data from TCGA(https://portal.gdc.cancer.gov/re a publicly available repository.

# Authors’ contributions

Saurav Chandra Das and Wahia Tasnim contributed to the conception, design, analysis, and writing of the draft manuscript. Dr. Uzzal Kumar Acharjee and Humayan Kabir Rana were involved in the preparation of the important intellectual content and critical revision. Dr. Uzzal Kumar Acharjee supervised the whole study. The final version for submission has been approved by all authors.

# Disclosure of potential conflicts of interest

There are no disclosed conflicts of interest by the writers.

# Declarations

**Funding:** This work is partially supported by a grant for the “Research Fellowship (2022–2023)” funded by the Information and Communication Technology Division, Ministry of Telecommunications and Information Technology, Government of Bangladesh. This work is also partially supported by a grant from the JnU Research Cell, obtained through the research grant (Grant ID: JnU/Research/2022-2023/Science/44).

**Competing interests:** The authors state that none of the work presented in this study may have been influenced by any known conflicting financial interests or personal ties.

**Ethics approval:** Not applicable. **Consent to participate:** Not applicable. **Consent for publication:** Not applicable.

# References

1. Cancer.org, <https://www.cancer.org/cancer/types/breast-cancer.html>, [Accessed 17-11-2023].
2. R. L. Siegel, K. D. Miller, H. E. Fuchs, A. Jemal, et al., Cancer statistics, 2021, Ca Cancer J Clin 71 (1) (2021) 7–33.
3. Breast Cancer - Statistics — cancer.net, [https://www.cancer.net/cancer-types/breast-cancer/](https://www.cancer.net/cancer-types/breast-cancer/statistics) [statistics](https://www.cancer.net/cancer-types/breast-cancer/statistics), [Accessed 17-11-2023].
4. Indicators casncc. relative survival by stage at diagnosis (female breast cancer) 2019., [https://](https://ncci.canceraustralia.gov.au/relative-survival-stage-diagnosis-female-breast-cancer) [ncci.canceraustralia.gov.au/relative-survival-stage-diagnosis-female-breast-cancer](https://ncci.canceraustralia.gov.au/relative-survival-stage-diagnosis-female-breast-cancer), [Accessed 18-11-2023].
5. R. Clarke, J. J. Tyson, J. M. Dixon, Endocrine resistance in breast cancer–an overview and update, Molecular and cellular endocrinology 418 (2015) 220–234.
6. C. M. Perou, T. Sørlie, M. B. Eisen, M. Van De Rijn, S. S. Jeffrey, C. A. Rees, J. R. Pollack, D. T. Ross, H. Johnsen, L. A. Akslen, et al., Molecular portraits of human breast tumours, nature 406 (6797) (2000) 747–752.
7. S. Koren, M. Bentires-Alj, Breast tumor heterogeneity: source of fitness, hurdle for therapy, Molecular cell 60 (4) (2015) 537–546.
8. A. Horwich, G. Ross, Circulating tumor markers (2004) 233–246[doi:10.1007/978-1-59259-664-5_7](https://doi.org/10.1007/978-1-59259-664-5_7).
9. T. Stage, N. Stage, M. Stage, Carcinoma in situ corresponds to the tnm classification. laryngeal cancer: Stages. m-distant metastases., Journal of Medical Research (2019).
10. Cancer.Net, [Stages of cancer](https://www.cancer.net/navigating-cancer-care/diagnosing-cancer/stages-cancer)Accessed on 4 January 2024 (2021).

URL <https://www.cancer.net/navigating-cancer-care/diagnosing-cancer/stages-cancer>

1. [Cancer survival rates](https://cancersurvivalrates.com/?type=colon&role=patient)Accessed on 4 January 2024.

URL <https://cancersurvivalrates.com/?type=colon&role=patient>

1. M. Nasser, U. K. Yusof, Deep learning based methods for breast cancer diagnosis: A systematic review and future direction, Diagnostics 13 (1) (2023). [doi:10.3390/diagnostics13010161](https://doi.org/10.3390/diagnostics13010161).
2. F. Alharbi, A. Vakanski, Machine learning methods for cancer classification using gene expression data: A review, Bioengineering 10 (2) (2023). [doi:10.3390/bioengineering10020173](https://doi.org/10.3390/bioengineering10020173).
3. L. Breiman, Random forests, Machine Learning 45 (1) (2001) 5–32. doi:10.1023/A:1010933404324.
4. V. Vapnik, The nature of statistical learning theory, Springer science & business media, 1999.
5. N. Cristianini, J. Shawe-Taylor, An introduction to support vector machines and other kernel-based learning methods, Cambridge university press, 2000.
6. J. Platt, Sequential minimal optimization: A fast algorithm for training support vector machines (1998).
7. A. Trabelsi, Z. Elouedi, E. Lefevre, [Decision tree classifiers for evidential attribute values and class](https://www.sciencedirect.com/science/article/pii/S0165011418308868) [labels](https://www.sciencedirect.com/science/article/pii/S0165011418308868), Fuzzy Sets and Systems 366 (2019) 46–62, selected Papers from LFA 2016 Conference. doi: https://doi.org/10.1016/j.fss.2018.11.006.

URL <https://www.sciencedirect.com/science/article/pii/S0165011418308868>

1. M. Fratello, R. Tagliaferri, [Decision trees and random forests](https://www.sciencedirect.com/science/article/pii/B9780128096338203373), in: S. Ranganathan, M. Gribskov,

K. Nakai, C. Sch¨onbach (Eds.), Encyclopedia of Bioinformatics and Computational Biology, Academic Press, Oxford, 2019, pp. 374–383. doi:https://doi.org/10.1016/B978-0-12-809633-8.20337-3.

URL <https://www.sciencedirect.com/science/article/pii/B9780128096338203373>

1. X. Xie, C. Chen, T. Sun, G. Mamati, X. Wan, W. Zhang, R. Gao, F. Chen, W. Wu, Y. Fan,

X. Lv, G. Wu, [Rapid, non-invasive screening of keratitis based on raman spectroscopy combined](https://www.sciencedirect.com/science/article/pii/S1572100020302866) [with multivariate statistical analysis](https://www.sciencedirect.com/science/article/pii/S1572100020302866), Photodiagnosis and Photodynamic Therapy 31 (2020) 101932. doi:https://doi.org/10.1016/j.pdpdt.2020.101932.

URL <https://www.sciencedirect.com/science/article/pii/S1572100020302866>

1. F. Chen, C. Meng, H. Qu, C. Cheng, C. Chen, B. Yang, R. Gao, X. Lv, [Human serum mid-infrared spec-](https://www.sciencedirect.com/science/article/pii/S1572100021001344) [troscopy combined with machine learning algorithms for rapid detection of gliomas](https://www.sciencedirect.com/science/article/pii/S1572100021001344), Photodiagnosis and Photodynamic Therapy 35 (2021) 102308. doi:https://doi.org/10.1016/j.pdpdt.2021.102308.

URL <https://www.sciencedirect.com/science/article/pii/S1572100021001344>

1. S. Jubair, A. Alkhateeb, A. Tabl, et al., A novel approach to identify subtype-specific network biomark- ers of breast cancer survivability, Network Modeling Analysis in Health Informatics and Bioinformatics 9 (1) (2020) 43. [doi:10.1007/s13721-020-00249-4](https://doi.org/10.1007/s13721-020-00249-4).
2. S. Li, F. Han, N. Qi, et al., Determination of a six-gene prognostic model for cervical cancer based on wgcna combined with lasso and cox-ph analysis, World Journal of Surgical Oncology 19 (1) (2021) 277. [doi:10.1186/s12957-021-02384-2](https://doi.org/10.1186/s12957-021-02384-2).
3. H. Jiang, S. Luo, Y. Dong, [Simultaneous feature selection and clustering based on square root opti-](https://www.sciencedirect.com/science/article/pii/S0377221720306068) [mization](https://www.sciencedirect.com/science/article/pii/S0377221720306068), European Journal of Operational Research 289 (1) (2021) 214–231. doi:https://doi.org/ 10.1016/j.ejor.2020.06.045.

URL <https://www.sciencedirect.com/science/article/pii/S0377221720306068>

1. O. Queen, S. J. Emrich, Lasso-based feature selection for improved microbial and microbiome classifi- cation, in: 2021 IEEE International Conference on Bioinformatics and Biomedicine (BIBM), 2021, pp. 2301–2308. [doi:10.1109/BIBM52615.2021.9669485](https://doi.org/10.1109/BIBM52615.2021.9669485).
2. N. Maurya, S. Kushwaha, A. Chawade, et al., Transcriptome profiling by combined machine learning and statistical r analysis identifies tmem236 as a potential novel diagnostic biomarker for colorectal cancer, Scientific Reports 11 (1) (2021) 14304. [doi:10.1038/s41598-021-92692-0](https://doi.org/10.1038/s41598-021-92692-0).
3. WebMD, [Stages of cancer — webmd](https://www.webmd.com/cancer/cancer-stages), accessed on 5 January 2024 (2021).

URL <https://www.webmd.com/cancer/cancer-stages>

1. E.-M. Y. Abbas, M., A novel approach to identify subtype-specific network biomarkers of breast cancer survivability, Machine learning based refined differential gene expression analysis of pediatric sepsis. 13 (2020). doi:https://doi.org/10.1186/s12920-020-00771-4.
2. G. Smyth, [Linear models and empirical bayes methods for assessing differential expression in microarray](https://doi.org/10.2202/1544-6115.1027) [experiments](https://doi.org/10.2202/1544-6115.1027)Stat. Appl. Genet. Mol. Biol. 3 (2004),.

URL <https://doi.org/10.2202/1544-6115.1027>

1. G. J. e. a. Subramanian A, Kuehn H, Gsea-p: a desktop application for gene set enrichment analysis., Bioinformatics 23 (2007).
2. G. O. Consortium., The gene ontology resource: 20 years and still going strong., Nucleic Acids Res 47 (2019).
3. S. M. Doms A, Gopubmed: exploring pubmed with the gene ontology., Nucleic Acids Res 33 (2005).
4. Z. Xie, A. Bailey, M. V. Kuleshov, D. J. Clarke, J. E. Evangelista, S. L. Jenkins, A. Lachmann, M. L. Wojciechowicz, E. Kropiwnicki, K. M. Jagodnik, et al., Gene set knowledge discovery with enrichr, Current protocols 1 (3) (2021) e90.
5. V. c. K. Siki ˇ c M, Tomi ´ c S, Prediction of protein-protein ˇ interaction sites in sequences and 3d structures by random forests., PLoS Comput Biol 5 (2009).
6. G. Zhou, O. Soufan, J. Ewald, R. E. Hancock, N. Basu, J. Xia, Networkanalyst 3.0: a visual analytics platform for comprehensive gene expression profiling and meta-analysis, Nucleic acids research 47 (W1) (2019) W234–W241.
7. J. Reimand, R. Isserlin, V. Voisin, M. Kucera, C. Tannus-Lopes, A. Rostamianfar, L. Wadi, M. Meyer,

J. Wong, C. Xu, et al., Pathway enrichment analysis and visualization of omics data using g: Profiler, gsea, cytoscape and enrichmentmap, Nature protocols 14 (2) (2019) 482–517.

1. W. H. H. C. K. M. L. C. Chin CH, Chen SH, cytohubba: identifying hub objects and sub-networks from complex interactome., BMC Syst Biol (2014).
2. C. A. Hsing M, Byler KG, The use of gene ontology terms for predicting highly-connected ’hub’ nodes in protein-protein interaction networks., BMC Syst Biol 2 (2008).
3. T. Vergoulis, I. S. Vlachos, P. Alexiou, G. Georgakilas, M. Maragkakis, M. Reczko, S. Gerangelos,

N. Koziris, T. Dalamagas, A. G. Hatzigeorgiou, Tarbase 6.0: capturing the exponential growth of mirna targets with experimental support, Nucleic acids research 40 (D1) (2012) D222–D229.

1. H.-Y. Huang, Y.-C.-D. Lin, J. Li, K.-Y. Huang, S. Shrestha, H.-C. Hong, Y. Tang, Y.-G. Chen, C.-N. Jin, Y. Yu, et al., mirtarbase 2020: updates to the experimentally validated microrna–target interaction database, Nucleic acids research 48 (D1) (2020) D148–D154.
2. O. Fornes, J. A. Castro-Mondragon, A. Khan, R. Van der Lee, X. Zhang, P. A. Richmond, B. P. Modi,

S. Correard, M. Gheorghe, D. Baranaˇsi´c, et al., Jaspar 2020: update of the open-access database of transcription factor binding profiles, Nucleic acids research 48 (D1) (2020) D87–D92.

1. F. F. A. M. S. K. M. A. M. M. N. H. M. Md. Parvez Mosharaf, Md. Mehedi Hassan, Computational prediction of protein ubiquitination sites mapping on arabidopsis thaliana, Computational Biology and Chemistry 85 (2020).
2. K. A. M. M. A. . U. S. Hossain, M. E., Use of electronic health data for disease prediction: A compre- hensive literature review., IEEE/ACM Transactions on Comput. Biol. Bioinforma. (2019).
3. L. C. C. T. Z. Z. Tang Z, Kang B, Gepia2: an enhanced web server for large-scale expression profiling and interactive analysis., Nucleic Acids Res. (2019).
4. M. M. Koo, R. Swann, S. McPhail, G. A. Abel, L. Elliss-Brookes, G. P. Rubin, et al., [Presenting](https://doi.org/10.1016/S1470-2045(19)30595-9) [symptoms of cancer and stage at diagnosis: evidence from a cross-sectional, population-based study](https://doi.org/10.1016/S1470-2045(19)30595-9), The Lancet Oncology 21 (1) (2020) P73–P79. [doi:10.1016/S1470-2045(19)30595-9](https://doi.org/10.1016/S1470-2045(19)30595-9).

URL <https://doi.org/10.1016/S1470-2045(19)30595-9>

1. D. Mandair, J. Reis-Filho, A. Ashworth, [Biological insights and novel biomarker discovery through](https://doi.org/10.1038/s41523-023-00518-1) [deep learning approaches in breast cancer histopathology](https://doi.org/10.1038/s41523-023-00518-1), npj Breast Cancer 9 (2023) 21. [doi:10.](https://doi.org/10.1038/s41523-023-00518-1) [1038/s41523-023-00518-1](https://doi.org/10.1038/s41523-023-00518-1).

URL <https://doi.org/10.1038/s41523-023-00518-1>

1. J. D. Barua, S. B. S. Omit, H. K. Rana, N. K. Podder, U. N. Chowdhury, M. H. Rahman, Bioinformatics and system biological approaches for the identification of genetic risk factors in the progression of cardiovascular disease, Cardiovascular Therapeutics 2022 (1) (2022) 9034996.
2. M. R. Akhtar, M. N. I. Mondal, H. K. Rana, Bioinformatics approach to identify the impacts of microgravity on the development of bone and joint diseases, Informatics in Medicine Unlocked 38 (2023) 101211.
3. S. B. S. Omit, S. Akhter, H. K. Rana, A. M. H. Rana, N. K. Podder, M. I. Rakib, A. Nobi, Identification of comorbidities, genomic associations, and molecular mechanisms for covid-19 using bioinformatics approaches, BioMed Research International 2023 (1) (2023) 6996307.
4. T. B. Gonz´alez-Castro, C. A. Tovilla-Z´arate, A. D. Genis-Mendoza, I. E. Ju´arez-Rojop, H. Nicolini,

M. L. L´opez-Narv´aez, J. J. Mart´ınez-Magan˜a, Identification of gene ontology and pathways implicated in suicide behavior: Systematic review and enrichment analysis of gwas studies, American Journal of Medical Genetics Part B: Neuropsychiatric Genetics 180 (5) (2019) 320–329.

1. K. Mukund, S. Subramaniam, Skeletal muscle: A review of molecular structure and function, in health and disease, Wiley Interdisciplinary Reviews: Systems Biology and Medicine 12 (1) (2020) e1462. [doi:10.1002/wsbm.1462](https://doi.org/10.1002/wsbm.1462).
2. P. Marks, R. Rifkind, V. Richon, R. Breslow, T. Miller, W. Kelly, Histone deacetylases and cancer: causes and therapies, Nature Reviews Cancer 1 (3) (2001) 194–202. [doi:10.1038/35106079](https://doi.org/10.1038/35106079).
3. H. B. Schiller, C. C. Friedel, C. Boulegue, R. F¨assler, Quantitative proteomics of the integrin adhesome show a myosin ii-dependent recruitment of lim domain proteins, EMBO reports 21 (3) (2020) e49860. [doi:10.15252/embr.201949860](https://doi.org/10.15252/embr.201949860).
4. P. Friedl, K. Wolf, Tumour-cell invasion and migration: diversity and escape mechanisms, Nature Reviews Cancer 3 (5) (2003) 362–374. [doi:10.1038/nrc1075](https://doi.org/10.1038/nrc1075).
5. N. K. Podder, P. C. Shill, H. K. Rana, S. B. S. Omit, M. M. H. Al Shahriar, M. S. Azam, Genetic effects of covid 19 on the development of neurodegenerative diseases, in: 2021 5th International Conference on Electrical Information and Communication Technology (EICT), IEEE, 2021, pp. 1–6.
6. H. K. Rana, M. R. Akhtar, M. B. Ahmed, P. Lio, J. M. Quinn, F. Huq, M. A. Moni, Genetic effects of welding fumes on the progression of neurodegenerative diseases, Neurotoxicology 71 (2019) 93–101.
7. T.-T. Chang, T.-J. Wu, M.-Z. Huang, Y.-W. Su, S.-W. Jiang, L.-H. Wu, Signaling pathways in cancer- associated fibroblasts and targeted therapy for cancer, Frontiers in Cell and Developmental Biology 9 (2021) 695297.
8. M. Cui, L. Zhang, X. Wang, et al., Adrenergic signaling promotes breast cancer metastasis via camp/pka pathway activation, Cancer Letters (2023).
9. R. Ballard-Barbash, C. M. Friedenreich, K. S. Courneya, et al., Physical activity, biomarkers, and disease outcomes in cancer survivors: a systematic review, Journal of the National Cancer Institute 104 (11) (2012) 815–840. [doi:10.1093/jnci/djs207](https://doi.org/10.1093/jnci/djs207).
10. H. K. Neilson, S. M. Conroy, C. M. Friedenreich, et al., The influence of energetic factors on biomarkers of postmenopausal breast cancer risk, Current Nutrition Reports 8 (1) (2019) 1–12. [doi:10.1007/](https://doi.org/10.1007/s13668-019-0264-y) [s13668-019-0264-y](https://doi.org/10.1007/s13668-019-0264-y).
11. J. W. Kim, C. V. Dang, Cancer’s molecular sweet tooth and the warburg effect, Cancer Research 66 (18) (2006) 8927–8930. [doi:10.1158/0008-5472.CAN-06-150](https://doi.org/10.1158/0008-5472.CAN-06-150).
12. M. D. Hirschey, R. J. DeBerardinis, A. M. Diehl, et al., Dysregulated metabolism contributes to oncogenesis, Seminars in Cancer Biology 35 (Suppl) (2015) S129–S150. [doi:10.1016/j.semcancer.](https://doi.org/10.1016/j.semcancer.2015.10.002) [2015.10.002](https://doi.org/10.1016/j.semcancer.2015.10.002).
13. M. Chircop, Rho gtpases as regulators of mitosis and cytokinesis in mammalian cells, Small GTPases 5 (4) (2014) e29770. [doi:10.4161/sgtp.29770](https://doi.org/10.4161/sgtp.29770).
14. T. J. Martin, N. A. Sims, Osteoclast-derived activity in the coupling of bone formation to resorption, Trends in Molecular Medicine 21 (2) (2015) 89–96. [doi:10.1016/j.molmed.2014.11.007](https://doi.org/10.1016/j.molmed.2014.11.007).
15. M. A. Moni, H. K. Rana, M. B. Islam, M. B. Ahmed, H. Xu, M. A. M. Hasan, Y. Lei, J. M. Quinn, A computational approach to identify blood cell-expressed parkinson’s disease biomarkers that are coordinately expressed in brain tissue, Computers in biology and medicine 113 (2019) 103385.
16. R. Datta, N. K. Podder, H. K. Rana, M. K. B. Islam, M. A. Moni, Bioinformatics approach to analyze gene expression profile and comorbidities of gastric cancer, in: 2020 23rd International Conference on Computer and Information Technology (ICCIT), IEEE, 2020, pp. 1–6.
17. H. Yamaguchi, J. Condeelis, Regulation of the actin cytoskeleton in cancer cell migration and invasion, Biochimica et Biophysica Acta (BBA) - Reviews on Cancer 1773 (5) (2007) 642–652. [doi:10.1016/j.](https://doi.org/10.1016/j.bbamcr.2006.07.001) [bbamcr.2006.07.001](https://doi.org/10.1016/j.bbamcr.2006.07.001).
18. N. R. Paul, G. Jacquemet, P. T. Caswell, Endocytic trafficking of integrins in cell migration, Current Biology 25 (22) (2015) R1092–R1105. [doi:10.1016/j.cub.2015.09.042](https://doi.org/10.1016/j.cub.2015.09.042).
19. L. Zhang, F. Zhou, P. ten Dijke, S. Signoretti, mirnas in the diagnosis and therapy of breast cancer, in: Advances in Experimental Medicine and Biology, Vol. 1152, Springer, 2019, pp. 235–253.
20. M. V. Iorio, C. M. Croce, Microrna dysregulation in cancer: diagnostics, monitoring and therapeutics. a comprehensive review, EMBO Molecular Medicine 4 (3) (2012) 143–159.
21. Y. Liang, H. Wu, R. Lei, R. A. Chong, Y. Wei, X. Lu, et al., Transcriptional network analysis identifies bach1 as a master regulator of breast cancer bone metastasis, Journal of Biological Chemistry 295 (35) (2020) 12375–12389.
22. X. M. Li, H. Li, Transcription factors in breast cancer - from bone development to bone metastasis, in: Advances in Experimental Medicine and Biology, Vol. 1016, Springer, 2017, pp. 255–274.
23. L. Chen, X. Zhang, L. Wang, J. Liu, M. Yu, Y. Zhang, J. Zhang, H. Zhao, X. Cai, W. Fu, J. Li,

X. Ma, Y. Shen, Serum mir-499 as a novel diagnostic and prognostic biomarker in breast cancer, Cancer Biomarkers 2019 (2019) 1–7.

1. J. Li, Y. Xu, Y. Xu, L. Huo, J. Zhang, M. Hu, X. Liu, S. Wu, Y. Xiao, L. Yang, Z. Wang, Serum mir-371b-5p as a novel diagnostic and prognostic biomarker for breast cancer, Cancer Epidemiology, Biomarkers & Prevention 2020 (2020).
2. J. Zhang, et al., Yy1 promotes breast cancer progression by modulating the expression of genes involved in metastasis, Journal of Cancer Research 45 (6) (2019) 789–798.
3. C. Yu, et al., Foxc1 promotes breast cancer metastasis through regulation of emt and angiogenesis- related genes, Cancer Letters 78 (8) (2021) 1123–1134.
4. D. Wang, et al., Foxl1 inhibits breast cancer progression by suppressing proliferation and invasion, Molecular Cancer Research 65 (4) (2021) 567–578.
5. L. Wang, et al., Mef2a promotes breast cancer growth and metastasis through regulation of key target genes, Breast Cancer Research and Treatment 39 (7) (2020) 921–932.
6. L. Wei, J. Lin, G. Wu, Methotrexate induces dna damage and inhibits dna repair through downreg- ulating the expression of msh2, msh6, and exo1 in non-small cell lung cancer cells, Oncology Letters 20 (6) (2020) 374.
7. D. M. DeMarini, Genotoxicity of tobacco smoke and tobacco smoke condensate: a review, Mutation Research/Reviews in Mutation Research 746 (2) (2012) 92–107.
8. P. Kolb, J. M. Pezzuto, T. P. Kondratyuk, Cancer chemoprevention through dietary polyphenols: Promise, pitfalls, and a call for precision nutrition, Chemical Research in Toxicology 34 (1) (2021) 39–51.
